# Risk Factors in Sporadic Early-Onset Colorectal Cancer, Current Evidence and Emerging Insights: A Systematic Review

**DOI:** 10.3390/cancers18101515

**Published:** 2026-05-08

**Authors:** Meghana Maddula, Jordan E. Cohen, Dulitha Kumarasinghe, Mandy L. Ballinger, Jaqueline L. E. Tearle, Kylie R. James, Adnan Nagrial, Megan Barnet, Subotheni Thavaneswaran

**Affiliations:** 1 Faculty of Medicine and Health, University of New South Wales, Sydney, NSW 2052, Australia; 2 Department of Anaesthesia, Westmead Hospital, Sydney, NSW 2145, Australia; 3 Centre for Molecular Oncology, University of New South Wales, Sydney, NSW 2052, Australia; 4 School of Biomedical Sciences, University of New South Wales, Sydney, NSW 2052, Australia; 5 Garvan Institute of Medical Research, Darlinghurst, Sydney, NSW 2010, Australia; 6 Department of Medical Oncology, Westmead Hospital, Sydney, NSW 2145, Australia; 7 The Kinghorn Centre, St Vincent’s Health Network Sydney, Sydney, NSW 2010, Australia; 8 NHMRC Clinical Trials Centre, The University of Sydney, Sydney, NSW 2050, Australia

**Keywords:** early-onset colorectal cancer, sporadic colorectal cancer, risk factors, systematic review

## Abstract

**Background:** The incidence of early-onset colorectal cancer (EOCRC) is rising globally, yet its underlying risk factors remain incompletely understood, particularly in cases without recognised hereditary syndromes. **Objectives:** To update and synthesise the current body of evidence on modifiable and non-modifiable risk factors for sporadic early-onset colorectal cancer. **Methods:** A systematic review of peer-reviewed articles published in English reporting original observational research examining risk factors for sporadic early-onset colorectal cancer (<50 years old) was conducted. PubMed and EMBASE databases were searched from inception to March 2025. Across studies, effect measures varied; therefore, the synthesis focused on the consistency of associations rather than the direct comparison of effect sizes. **Results:** The initial search identified 2575 papers; 34 studies were included after screening. Several consistent associations were identified, with dietary and lifestyle factors along with metabolic conditions emerging as key risk factors. EOCRC risk was higher in males (adjusted odds ratio (aOR) 1.36–2.21 across studies), individuals of Caucasian ethnicity (aORs 1.48–2.56), and in individuals whose age was approaching 50 years (per year, aORs 1.05–1.11). Putatively sporadic EOCRC was associated with a family history of CRC or other cancers (aORs up to 8.61). Other key factors linked to higher risk included obesity (aORs 1.92–2.88; adjusted Hazard Ratios (aHRs) 1.04–1.82), metabolic syndrome (aORs 1.25–2.48; aHRs 1.2–1.26), diabetes (aORs 1.24–3.42), Western dietary patterns (aORs 1.84–2.99), and sedentary behaviours (adjusted relative risks (aRR) 1.69–2.44). Moderate-to-vigorous exercise appeared protective (aORs 0.34–0.58), as did higher vitamin D levels (aHRs 0.41–0.61). Evidence for smoking, alcohol, medications and early/in utero environmental exposures was inconsistent. **Conclusions:** Lifestyle and metabolic factors, including Western dietary patterns, obesity and sedentary behaviours, were associated with sporadic EOCRC. Family history also emerged as a significant contributor to putatively sporadic disease, suggesting heritable influences beyond recognised syndromes and interplay between environmental factors and genetic predisposition. Future research should focus on integrated tumour and germline profiling, including broader genomic analyses in well-characterised cohorts, to better understand potential pathogenic mechanisms and support the development of risk stratification approaches.

## Simple Summary

Colorectal cancer is a major cause of cancer-related death worldwide and is increasingly being diagnosed in individuals under 50 years of age, when it is referred to as early-onset colorectal cancer. While a minority of cases are explained by recognised hereditary syndromes, most occur without an identified genetic cause and are considered sporadic. This suggests that some cases may be due to unrecognised or currently undefined hereditary syndromes, as well as environmental and lifestyle factors, and the interaction between them. In this systematic review, we synthesise current research to better understand potential risk factors and contributors. By identifying consistent patterns, our findings aim to improve understanding of the rising incidence of early-onset colorectal cancer and support the development of screening and prevention strategies and future research to identify underlying causes.

## 1. Introduction

Colorectal cancer (CRC) is a leading cause of cancer-related mortality and morbidity worldwide [1,2,3]. Recent data from The International Agency for Research on Cancer reported colorectal cancer to be the third most common cancer globally and the second leading cause of cancer-related death, with the highest incidence in Australia and New Zealand [3,4]. The global burden of disease is projected to increase by 63% to over 3 million new cases and almost 2 million deaths by 2040 [4,5]. Traditionally considered a disease of older adults, colorectal cancer incidence over the past two decades has stabilised or declined in those 50 years and over, in contrast to a rise in people under 50 [5]. This emerging cohort is termed ‘early-onset colorectal cancer’ (EOCRC), a widely recognised entity with recent formalisation in the Delphi Initiative for early-onset colorectal cancer (DIRECt) consensus guidelines [6]. Concerningly, patients with EOCRC are more likely to present with advanced disease and have more aggressive disease characteristics when compared with average-onset cases [7,8,9,10,11]. In Australia, the incidence of EOCRC has risen by almost 50% between 2000 and 2024 [12], one of the highest age-standardised increments globally [5,13,14,15,16]. Although EOCRC represents a minority of colorectal cancer diagnoses, its rising incidence is clinically significant given the substantial associated morbidity and disproportionate loss of productive life years. Moreover, the trend may reflect generational shifts in risk exposures that could influence future population-level disease patterns [7,8,9,10,11,12].

Hereditary cancer syndromes such as Lynch syndrome and Familial Adenomatous Polyposis are well-established risk factors for colorectal cancer diagnoses; however, they account for less than 30% of EOCRC cases [17,18,19,20,21]. The majority of EOCRC cases are termed ‘sporadic’, defined as cases occurring in the absence of a recognised hereditary cancer syndrome (e.g., Lynch syndrome) or known monogenic cause [17,18,19,20,21]. This categorisation reflects significant heterogeneity in pathogenesis, combining both cases resulting from unrecognised hereditary predisposition and/or shared familial, metabolic and environmental risk factors.

A wide range of factors has been implicated in the development of EOCRC, including lifestyle and dietary exposures, metabolic risk factors and comorbidities [22,23,24,25,26,27,28]. The most recent systematic reviews by Hua et al. 2023 [25] and Ye et al. 2023 [27] examined risk factors associated with EOCRC or adenoma development across 36 and 28 eligible studies, respectively, highlighting the relatively limited evidence base. In line with Cochrane recommendations to update reviews every 2–3 years [29], and considering the evolving evidence, including 14 new studies published since previous analyses, we conducted a systematic review of the current literature in EOCRC with a focus on sporadic cases.

## 2. Methods

### 2.1. Search Strategy

A systematic review was conducted to identify risk factors associated with sporadic EOCRC. A search strategy developed in consultation with an academic librarian at the University of New South Wales (UNSW) was applied to PubMed and EMBASE databases from inception to 1 March 2025. Search terms included ‘early-onset’, ‘young-onset’, ‘colorectal cancer’ and ‘risk-factors’. The search strategy was intentionally designed to prioritise sensitivity using broad exposure-related terms, given the heterogeneity and evolving nature of EOCRC risk factor research, and to avoid restricting the search to pre-specified exposures. Detailed search strategies are outlined in the Supplementary Materials. Review articles were excluded, but their references were screened for additional studies. The review was prospectively registered [PROSPERO (CRD420251063020)]. PRISMA Checklists have been included in the Supplementary Materials [30].

### 2.2. Eligibility

Inclusion criteria were as follows: (1) published in full-text format in English and peer-reviewed journals; (2) original observational study; focused on (3) sporadic-onset colorectal cancer in the <50-year-old population and (4) evaluation of risk factors. Studies including mixed-age populations were eligible if age-stratified analyses for individuals < 50 years were reported or extractable. Studies were required to primarily evaluate sporadic EOCRC, defined for the purposes of this review as cases occurring in the absence of recognised hereditary cancer syndromes. Studies were included if they explicitly excluded hereditary syndromes, or where explicit exclusion of hereditary syndromes was not stated, studies were eligible if appropriate adjustment for family history or sensitivity analyses were undertaken. Detailed eligibility criteria are provided in the Supplementary Materials. Screening, data extraction and narrative synthesis were conducted between March and July 2025.

### 2.3. Study Selection, Quality Assessment, and Data Extraction

Abstract screening was conducted by M.M. and D.K between March and April 2025. Two reviewers (M.M., J.E.C.) independently conducted full-text reviews between April and June 2025 with disagreements mediated by a third reviewer (M.B. or S.T.).

For cohort and case–control studies, study quality was evaluated by using the Newcastle–Ottawa Scale (NOS) [31]. For cross-sectional studies, a modified NOS was applied in line with published methodology [32]. Assessment focused on all other domains, including sample representativeness, exposure and outcome ascertainment, adjustment for key confounders and appropriateness of statistical analyses. In accordance with commonly used NOS interpretation, studies scoring ≥6 were considered to be of moderate-to-high methodological quality and were included in the final synthesis. This threshold was selected to ensure inclusion of studies with adequate methodological rigour while also capturing the breadth of available evidence in this emerging research space.

Data, including study characteristics, population demographics, risk factors examined, and key findings, were extracted by two reviewers (M.M., J.E.C.) using a predefined data collection template between May and June 2025.

### 2.4. Data Synthesis

Risk factors were organised using thematic categorisation. Initial categories were predefined based on established colorectal cancer risk factor domains such as lifestyle, metabolic and genetic factors and were further refined following full-text review. Two reviewers (M.M., J.E.C.) independently assigned exposures to thematic groups, with discrepancies resolved by consensus (M.B. or S.T.).

Within each category, findings were synthesised descriptively, accounting for study design, sample size, effect estimates and quality assessment scores to contextualise the strength and consistency of evidence. Data extraction results are provided in full in the Supplementary Materials.

Substantial methodological heterogeneity precluded meaningful quantitative pooling for meta-analysis. Instead, data were synthesised and reported in accordance with the SWiM (Synthesis without meta-analysis) reporting guidelines [33]. In line with SWiM principles, studies were grouped by exposure domain, and findings were synthesised using direction-of-effect comparisons and structured tabulation of adjusted effect estimates.

Variable effect measures (OR, HR, RR) were interpreted according to the direction and relative magnitude of associations. Direct numerical comparisons were avoided due to differences in underlying measures; instead, synthesis focused on consistency and direction of associations. Effect estimates are reported as odds ratios (ORs) or hazard ratios (HR), with ‘a’ denoting adjusted estimates (aOR, aHR) where applicable. Given the absence of formal meta-analysis, studies were not quantitatively weighted; however, the interpretation of findings was informed by study design, sample size and the extent of covariate adjustment. Greater emphasis was placed on larger, population-based cohort studies and those with comprehensive adjustment for key confounders. The quality of covariate adjustment was defined by the inclusion of major variables such as age, sex, family history and relevant lifestyle or metabolic factors where applicable and was determined by consensus from two reviewers (M.M. and J.E.C.).

Thematic categorisation was used not only to group exposures but also to facilitate structured comparison across studies, enabling evaluation of the consistency, direction, and relative strength of associations across clinically relevant domains. This approach supported the identification of recurring patterns, as well as areas of heterogeneity, across the evidence base.

## 3. Results

### 3.1. Study Characteristics

**Figure 1:**
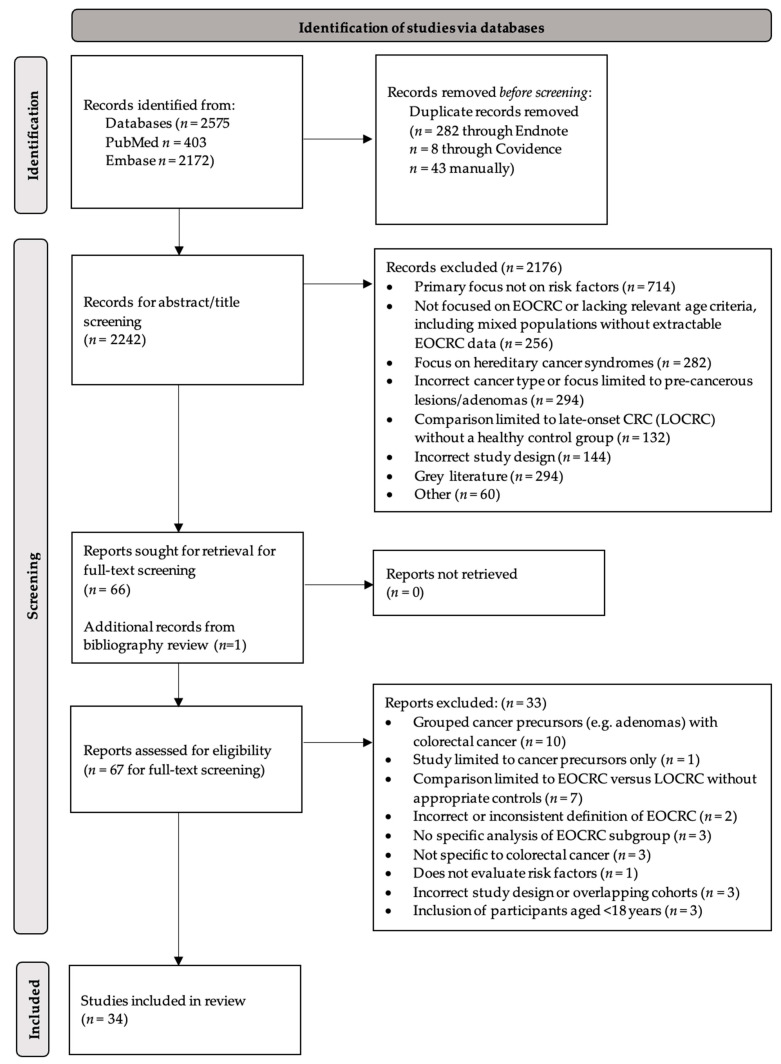
PRISMA diagram.

PubMed and EMBASE searches identified 2575 studies, with 2242 papers remaining after duplicate removal. Following screening, 67 papers were included for full-text review, and 34 were selected for final synthesis in accordance with the Preferred Reporting Items for Systematic Reviews and Meta-Analyses (PRISMA) guidelines (Figure 1). Studies included were of ‘good quality’ (score ≥ 6) (Supplementary Material Table S1), although methodological limitations varied across studies, particularly in relation to exposure measurement, confounder adjustment, and study design. Risk factors were analysed using thematic categorisation, with individual exposures grouped into broader domains of modifiable and non-modifiable factors and further organised into clinically relevant subcategories. All included studies were observational, and findings were therefore interpreted as associations and considered hypothesis-generating rather than definitively causal.

A summary of study characteristics is provided in Table 1, including key findings; effect estimates with more detailed information, including quality assessments, are reported in Supplementary Tables S1 and S2. 

### 3.2. Demographic Factors

Among individuals under 50 years, EOCRC risk was highest among those aged 40–49. Two large nationwide observational studies demonstrated a consistent age-related gradient, with risk increasing incrementally per additional year of age below 50. Effect estimates per additional year of age demonstrated a consistent direction across studies, with increasing age associated with higher EOCRC risk. Reported estimates ranged from an adjusted odds ratio (aOR) of 1.05 (95% CI 1.03–1.07) in Low et al. (*n* = 651 cases) [41] to an aOR of 1.11 (95% CI 1.08–1.14) in Zhang et al. (*n* = 156 cases) [67].

Eight studies examined gender, including large registry-based studies (Danial et al. [37], Glover et al. [40], Low et al. [41], Syed et al. [63]), a single institution case–control study (Gausman et al. 2020 [38]), a cross-sectional analysis (Zhang et al. [67]) and two single-centre cohort studies (Agazzi et al. [49], O’Sullivan et al. [59]). Registry-based case–control datasets consistently reported increased EOCRC risk among males, across differing effect estimates: Danial et al. (*n* = 13,800 cases); aOR 1.36 95% CI 1.32–1.39 [37], Gausman et al. 2020 (*n* = 269 cases); aOR 1.87 95% CI 1.39–2.51 [38], Low et al. (*n* = 651 cases); aOR 2.21 95% CI 1.68–2.91 [41], Syed et al. (*n* = 5710 cases); aOR 1.34 95% CI 1.27–1.41 [63]. Glover et al. (*n* = 1680 cases) [40] reported a positive association for male sex in univariate analysis (crude OR 1.29, 95% CI 1.17–1.41); however, this association was not retained after multivariate adjustment (aOR 0.27, 95% CI 0.25–0.30). In contrast, smaller single-centre and cross-sectional studies did not demonstrate a significant association between sex and EOCRC risk: Agazzi et al. (*n* = 27 cases) [49], crude OR 0.59, 95% CI 0.27–1.20; O’Sullivan et al. (*n* = 98 cases) [59], adjusted Hazard Ratio (aHR) 1.02, 95% CI 0.64–1.64, and Zhang et al. [67], aOR 0.83, 95% CI 0.54–1.26.

Ethnicity was evaluated in three US-based studies. Both registry-based studies reported increased EOCRC risk among Caucasian individuals compared to other ethnicities, including African American and Asian: Glover et al. (*n* = 1680 cases) aOR 2.56, 95% CI 2.43–2.69 [40], Syed et al. (*n* = 5710) aOR 1.48 95% 1.40–1.57 [63]. A cross-sectional analysis conducted by Zhang et al. [67] additionally reported a comparatively lower risk in Hispanic (aOR 0.43, 95% CI 0.22–0.84) and non-Hispanic Asian populations (aOR 0.38, 95% CI 0.16–0.92).

#### 3.2.1. Family History and Genomics

Family history was evaluated in nine studies, with all showing elevated EOCRC risk [35,37,38,40,44,59,60,63]. Registry-based datasets demonstrated the strongest positive associations, although effect measures and sizes varied. Syed et al. (*n* = 5710 cases) [63] reported markedly increased risk for individuals with a family history of CRC (aOR 28.67, 95% CI, 26.64–30.86) or of any history of gastrointestinal cancer (aOR 11.66, 95% CI, 10.97–12.39). Danial et al. (*n* = 13,800 cases) [37] reported an aOR 17.781 (95% CI, 16.974–18.627) for family history of CRC and Glover et al. (*n* = 1680 cases) [40] reported increased risk for individuals with a first-degree relative with CRC (aOR 8.61, 95% CI 4.83–15.75) or any cancer (aOR 7.33, 95% CI 6.18–8.70) after exclusion of known genetic syndromes. Moderate associations were also reported in smaller studies, with adjusted effect estimates ranging from aOR 2.37–8.61 for individuals with a family history of CRC (Gausman et al. 2020 *n* = 269 cases [38]; Collatuzzo et al. *n* = 189 cases [36]; Chang et al. 2021 *n* = 175 cases [35]; O’Sullivan et al. *n* = 98 cases [59]).

Agazzi et al. (*n* = 27 cases) [49] reported no significant association (OR 0.76, 95% CI 0.22–2.58), although this finding was based on only three cases with a family history of cancer and adjustment for age as the sole confounder.

Telomere Length

A single small case–control study (*n* = 70 cases) conducted by Martel et al. [42] examined telomere length as a potential marker of biological ageing in sporadic EOCRC. Shorter leukocyte telomere length was observed in cases compared with controls (122 kb vs. 292 kb, *p* < 0.001). No pathogenic variants in telomere maintenance genes were identified.

#### 3.2.2. Reproductive Factors

Reproductive factors were assessed in two studies. In a population-based case–control study, Chang et al. (*n* = 175 cases) [35] reported reduced EOCRC risk with higher parity (≥3 children vs. nulliparous, aOR 0.29, 95% CI 0.11–0.76). In contrast, a smaller prospective cohort study, O’Sullivan et al. (*n* = 98 cases) [59], did not demonstrate significant associations (aHR 0.76, 95% CI 0.34–1.73). However, comparisons across studies are limited by differences in study design and effect measure.

### 3.3. Lifestyle, Diet and Metabolic Risk Factors

#### 3.3.1. Diet

Seven studies examined dietary exposures in relation to EOCRC risk, with most demonstrating positive associations for Western dietary patterns and sugar-sweetened beverage (SSB) intake, while findings for individual food groups were heterogeneous. Direct comparisons between studies, however, were precluded by variability in effect measures.

In a Canadian population-based case–control study (*n* = 170 cases), Chang et al. [35] reported increased EOCRC risk with Western dietary patterns, characterised by higher intake of meat, sugary foods, and lower fibre intake (aOR 1.92, 95% CI 1.01–3.66). Similarly, in the prospective Nurses’ Health Study II (NHS II, *n* = 111 cases), Yue et al. [65] found that a higher empirical lifestyle index for hyperinsulinemia/hyperinsulinaemic diet (a validated score derived from a weighted sum of 12 food groups, BMI and physical activity) was associated with EOCRC (adjusted Hazard Ratio (aHR) 1.86, 95% CI 1.12–3.07).

Higher red and fresh meat intake was associated with increased EOCRC risk in an Iranian case–control study (*n* = 189 cases), with intake > 25.6 g/day compared to <12.83 g/day associated with increased risk (aOR 1.84, 95% CI 1.19–2.86) [36]. Supporting this direction of effect, a single institution Italian case–control study (*n* = 47 cases) reported higher median fresh meat intake among EOCRC cases (4–5 servings/week) compared with controls (2–3 servings/week) (*p* = 0.001), as well as higher processed meat intake (4–5 vs. 2–3 servings/week, *p* < 0.001), although effect estimates were not provided [44]. However, findings were not consistent across other cohorts. In a large Chinese prospective cohort (China Kadoorie Biobank, *n* = 222 cases), Pan et al. [60] found no association between red meat (daily vs. never, aHR 0.91 95% CI 0.53–1.57), poultry (daily vs. never, aHR 1.04 95% CI 0.66–1.65), or egg consumption (daily vs. never, aHR 0.57 95% CI 0.28–1.16) and EOCRC risk, although more frequent fish consumption (monthly vs. never, aHR 1.81, 95% CI 1.12–2.91) was associated with increased risk. Similarly, Chang et al. 2021 [35] (*n* = 175 cases) found no statistically significant association for higher red meat (≥5 vs. <2 servings/week aOR 1.06 95% CI 0.56–1.98), or processed meat intake (≥3 vs. <1 servings/week aOR 1.23 95% CI 0.62–2.42).

Findings for fruit and vegetable intake were inconsistent, with heterogeneity in both direction and magnitude of association as well as effect measure. In an Iranian case–control study (*n* = 189 cases), Collatuzzo et al. observed a protective effect with higher vegetable intake (422–576 g/day vs. <422 g/day, aOR 0.58, 95% CI 0.38–0.91) but increased risk with higher fruit intake (>429 g/day vs. <273 g/day, aOR 3.67, 95% CI 1.02–13.2) in a subgroup of individuals (<35 years, *n* = 34 cases) [36]. In contrast, a prospective Canadian cohort study by O’Sullivan et al. [59] (*n* = 98 cases) found no significant association between higher fruit and vegetable intake and EOCRC risk (8 servings/day vs. none: aHR 0.79, 95% CI 0.43–1.43). Similarly, Chang et al. [35] (*n* = 175 cases) reported no significant association for higher fruit and vegetable intake (≥6 vs. <3 servings/day: aOR 0.58, 95% CI 0.30–1.13).

EOCRC was also associated with intake of sugar-sweetened beverage (SSB) in both Chang et al. 2021 (≥7/week vs. <1/ week, aOR 2.99, 95% CI 1.57–5.68) [35] and Hur et al. utilising the NHIS II cohort (*n* = 109 cases) (≥2/day vs. <1 week, adjusted Risk Ratio (aRR) 2.18, 95% CI 1.10–4.35) [51].

Vitamin D

Three cohort studies (total *n* = 529 cases) evaluated vitamin D serum level or intake in relation to EOCRC risk and consistently reported inverse associations [36,55,56]. Higher serum levels (25–50 nmol/L and >50 nmol/L vs. <25 nmol/L; aHR 0.61, 95% CI 0.43–0.86 and aHR 0.41 95% CI 0.27–0.63, respectively) were associated with reduced risk [56], and higher intake was found to be protective (females with ≥450 IU/day vs. <300 IU/day, aHR 0.49, 95% CI 0.26–0.93/ever vs. never users, aOR 0.52, 95% CI 0.27–0.97) [36,55]. This association appeared more consistent for dietary vitamin D intake than for supplement use, with ≥300 IU/day vs. <300 IU/day associated with lower risk (aHR 0.50, 95% CI 0.27–0.93), while no significant association was observed for vitamin D supplementation (≥300 IU/day vs. <300 IU/day; aHR 0.64, 95% CI 0.28–1.45); sunlight exposure was not evaluated [55]. Kim et al. 2023 showed a dose–response (aHR 0.34 95% CI 0.15–0.79) within the Nurses’ Health Study II (NHS II) cohort, with no effect observed in those over 50 years [55].

#### 3.3.2. Physical Activity

Five studies (*n* = 594 cases) examined physical activity as an independent exposure.

Two population-based cohorts demonstrated increased EOCRC risk with sedentary behaviours, with consistent direction of association across studies despite differences in effect measures. In a Canadian case–control study, Chang et al. [35] (*n* = 175 cases) reported higher EOCRC risk among individuals with ≥10 h/day of sedentary time compared with <5 h/day (aOR 1.93, 95% CI 1.02–3.65). Similarly, in the NHIS cohort, Nguyen et al. [58] (*n* = 118 cases) found that >14 h/week of television viewing was associated with increased EOCRC risk (aRR 1.69, 95% CI 1.07–2.67). Subgroup analyses indicated stronger associations for rectal cancer (aRR 2.44 95% CI 1.03–5.78), in individuals without a family history of CRC (aRR 1.83 95% 1.15–2.95) and among those with BMI ≥ 25 kg/m^2^ (aRR 2.42 95% CI 1.4–4.11).

Consistent with these findings, higher levels of physical activity appeared protective in some analyses. Using NHIS data (*n* = 156 cases), Zhang et al. reported reduced EOCRC risk among individuals engaging in regular moderate (aOR 0.58 95% CI 0.34–0.99) and vigorous exercise (aOR 0.34 95% CI 0.34–0.99) [58]. In contrast, O’Sullivan et al. [59] found no significant association across increasing tertiles of metabolic equivalent hours per week in a small cohort study (*n* = 98 cases) (medium: aHR 1.15 95% CI 0.54–2.45, high: aHR 1.18 95% CI 0.70–1.8) and Puzzono et al. [44] reported no significant differences in activity frequency (<1/week, 1/week, >1/week) in a small case–control study (*n* = 47 cases) (*p* = 0.76, no effect measures reported).

#### 3.3.3. Metabolic Syndrome

Six large registry-based studies (total *n* = 39,213 cases) evaluated metabolic syndrome or clustered metabolic conditions (defined in Table 2) and consistently reported increased EOCRC risk; however, effect estimates were derived from both case–control and cohort study designs and therefore were not directly compared.

Jin et al. 2022 [53] (*n* = 8320 cases) (aHR 1.20, 95% CI 1.14–1.27) and Chen et al. 2021 [45] (*n* = 4673 cases) (aOR 1.25, 95% 2.09–2.43) both noted increased EOCRC risk in the presence of metabolic syndrome, with additive risk attributed to the number of metabolic risk factors (*p* < 0.001). Lundqvist et al. [48] (*n* = 2626 cases) reported a two-fold increase in EOCRC risk in individuals with metabolic disease (aHR 1.82, 95% 1.67–1.99), whilst Jimba et al. [52] (*n* = 1884 cases) found this increased risk primarily among men aged 20–49 years (aHR 1.26, 95% CI 1.05–1.50).

Studies examining clustered metabolic conditions (obesity, diabetes, hypertension, and markers of hepatic steatosis, including BMI, waist circumference, triglycerides and serum GGT) reported similar patterns. Park et al. [61] (*n* = 7910 cases) and Danial et al. [37] (*n* = 13,800 cases) reported increased EOCRC risk with elevated Fatty Liver Index Score (aHR 1.14, 95% CI 1.05–1.22) and combined metabolic conditions (obesity, diabetes, hypertension) (aOR 2.48, 95% CI 2.40–2.57), respectively.

#### 3.3.4. Obesity

Twelve studies examined weight-related metrics and EOCRC risk.

USA and Canadian Studies

Most studies assessing obesity were conducted in the USA and Canada. Analyses of the IBM Explorys database reported positive associations between obesity (BMI ≥ 30 kg/m^2^) and EOCRC across multiple studies, including Syed et al. [63] (2012–2016, 25–49 yo, *n* = 5710 cases) (aOR 2.88, 95% CI 2.74–3.04), Glover et al. [40] (2013–2018, 20–40 yo, *n* = 1680 cases) (aHR 1.82 95% CI 1.61–2.04), and Elangovan et al. [66] (2017–2021, 20–49 yo, *n* = 16,090 cases), noting variation in effect measures across studies. Elangovan et al. further reported age- and sex-specific gradients, with the stronger associations observed among women aged 20–39 and men aged 40–49 [66] (aOR 20–39 M: 1.92 (1.85–1.99), F: 2.22 (1.84–2.43)/40–49 (M: 1.96 (1.87–2.06), F: 1.49 (1.41–1.57)). In the NHS II cohort study, Liu et al. [57] also reported increased EOCRC risk with obesity (aRR 1.93, 95% CI 1.15–3.25), a 20% increase in risk per 5-unit increase in BMI (aRR 1.20 95% CI 1.05–1.38) and a two-fold increase among women gaining over 40 kg since age 18 (aRR 2.15 95% CI 1.01–4.55) [57].

In contrast, two case–control studies reported inverse or null associations between elevated BMI and EOCRC risk. Chang et al. [35] observed reduced risk among overweight (BMI 25–29.9 kg/m^2^) individuals (aOR 0.57, 95% CI 0.34–0.94). Low et al. showed reduced risk for both overweight and obese individuals at initial colonoscopy (aOR 0.69 95% CI 0.55–0.87; aOR 0.69 95% CI 0.55–0.86, respectively). However, in the Low et al. cohort, post hoc analyses suggested greater pre-diagnostic weight loss among EOCRC cases compared with controls in the five years preceding baseline colonoscopy [41].

Smaller studies, including Gausman et al. [38] (aOR 0.98, 95% CI 0.95–1.00), O’Sullivan et al. [59] (aHR 0.96 95% CI 0.58–1.57), and Zhang et al. [67] (aOR 1.04 95% CI 0.64–1.68), reported no significant associations.

East Asian Studies

Similar findings were reported in East Asian cohorts (total *n* = 16,034 cases), with broadly consistent direction of association despite differences in effect measures. Jin et al. 2022 [53] (*n* = 8320 cases) reported graded increases in EOCRC risk across BMI categories (23.0–24.9 kg/m^2^: aHR 1.10 95% CI 1.04–1.17, 25.0–29.9 kg/m^2^: aHR 1.19 95% CI 1.32–1.25 and ≥30 kg/m^2^: aHR 1.45 95% CI 1.31–1.61), with elevated risk also observed for central adiposity (≥100 cm in men, ≥95 cm in women) (aHR 1.53 95% CI 1.34–1.74). Song et al. [62] (*n* = 7492 cases) similarly reported increased EOCRC risk associated with persistent obesity (BMI ≥ 25 kg/m^2^) and persistent central adiposity (>90 cm in men, >85 cm in women) across two sequential health examinations in 2009 and 2011 (aHR 1.19, 95% CI 1.09–1.30) [62]. In a mainland Chinese cohort, Pan et al. [60] (*n* = 222 cases) observed a modest association between higher BMI (≥28 kg/m^2^) and EOCRC risk (aHR 1.04 95% CI 1.00–1.08), with effect estimates close to the null and therefore requiring cautious interpretation.

#### 3.3.5. Diabetes and Hyperglycaemia

Eight studies evaluated the association between diabetes and EOCRC, comprising large US-based datasets (Explorys *n* = 1680 cases [40,66], MarketScan *n* = 6001 cases) [47], nationwide Korean cohorts (NHIS *n* = 8320 cases [53], China Kadoori Biobank *n* = 222 cases [60]) and smaller institutional and prospective cohorts.

Two large US-based datasets specifically evaluated type 2 diabetes mellitus (T2DM). Li et al. [47] (*n* = 6001 cases) reported elevated risk overall (aOR 1.24, 95% CI 1.09–1.41), with the greatest risk observed in uncontrolled (aOR 1.59 95% CI 1.08–2.35) and complicated (aOR 1.57 95% CI 1.12–2.17) disease. Elangovan et al. [66] (*n* = 1680 cases) found that T2DM only increased EOCRC risk in men, with the strongest association seen in young men (20–39 years aOR 3.42 95% CI 2.85–5.37, 40–49 aOR 2.00 95% CI 1.75–2.28).

Several other large registry-based studies did not distinguish diabetes subtype but are considered in the context that T2DM represents 60–80% of diabetes in adults under 50 [68,69,70]. In the Explorys database, Glover et al. [40] noted a nearly 20-fold increase in EOCRC risk in those with diabetes (aOR 19.8, 95% CI 18.15–21.96). In the China Kadoorie Biobank study, Pan et al. [60] also noted an increased risk overall (aHR 2.20, 95% CI 1.08–4.49), with significant associations in women (aHR 2.62, 95% CI 1.06–6.48), but not men (aHR 1.80, 95% 0.56–5.76). In a nationwide Korean cohort, Jin et al. [53] also reported a small but significant increase in EOCRC risk (aHR 1.08, 95% CI 1.03–1.13).

In contrast, smaller regional and institutional cohorts reported no significant associations; O’Sullivan et al. [59] (aHR 0.90, 95% CI 0.28–2.93), Gausman et al. 2020 [38] (EOCRC 7% vs. controls 6%, *p* = 0.48, univariate analysis), and Chang et al. [35] (aOR 1.75 95% CI 0.57–0.98).

#### 3.3.6. Hypertension

Hypertension or elevated blood pressure was evaluated as an independent exposure in six studies (total EOCRC cases *n* = 30,709 cases).

In large registry-based datasets, hypertension was positively associated with EOCRC. Syed et al. [63] (*n* = 5710 cases) reported an aOR of 2.86 (2.70–3.03) and Elangovan et al. [66] (*n* = 16,090 cases) observed increased risk of EOCRC across age and sex, with the strongest associations seen among men aged 20–39 years (aOR 3.43, 95% CI 2.77–4.22). Jin et al. 2022 [53] (*n* = 8320 cases) reported increased risk with elevated blood pressure in a Korean cohort (aHR 1.13, 95% CI 1.07–1.18). Similarly, in the China Kadoorie Biobank, Pan et al. [60] (*n* = 222 cases) demonstrated an association between self-reported hypertension and EOCRC (aHR 1.99, 95% CI 1.04–3.81), with the greatest effect seen in women (aHR 2.32, 95% CI 1.01–5.34).

Conversely, no significant associations were observed in smaller studies; Gausman et al. [38] (case–control *n* = 269 cases, EOCRC 19% vs. controls 20%, *p* = 0.85, univariate analysis) and O’Sullivan et al. [59] (cohort; *n* = 98 cases, aHR 1.10 95% CI 0.59–2.08).

#### 3.3.7. Dyslipidaemia

Five studies examined dyslipidaemia, including hyperlipidaemia and hypertriglyceridaemia.

In two nationwide Korean NHIS cohorts, hypertriglyceridaemia was independently associated with EOCRC. Chang et al. 2024 [50] (2009–2011, *n* = 7492 cases) demonstrated that hypertriglyceridaemia (≥150 mg/dL) was associated with elevated EOCRC risk (aHR 1.10, 95% CI 1.03–1.17), with a dose–response relationship (highest quartile ≥ 161 mg/dL vs. lowest quartile < 71 mg/dL, *p* < 0.0001). Similarly, Jin et al. 2022 (2009–2019, *n* = 8320 cases) [53] reported an independent association between hypertriglyceridaemia and EOCRC (aHR 1.13, 95% CI 1.08–1.18).

In large USA-based studies (total *n* = 21,800 cases), hyperlipidaemia was also associated with EOCRC. Syed et al. [63] (*n* = 5710 cases) reported aOR 2.39 (95% CI 2.23–2.55), and Elangovan et al. [66] (*n* = 16,090 cases) noted elevated risk across age and gender, with the strongest association seen among men aged 20–39 years (aOR 3.42, 95% CI 2.48–4.04). Conversely, in a single institution case–control study (*n* = 269 cases), Gausman et al. 2020 [38] found the reverse, that dyslipidaemia appeared protective (aOR 0.57, 95% 0.38–0.83), noting no differences in BMI and metabolic syndrome between cases and controls.

#### 3.3.8. Alcohol

Eight studies examined alcohol consumption, with findings varying by population and exposure definition. Two large US-based case–control analyses utilised the IBM Explorys dataset: Danial et al. [37] (*n* = 13,800 cases) and Syed et al. [63] (*n* = 5710 cases) reported positive associations between alcohol use and EOCRC risk (aOR 1.90 95% CI 1.78–2.02 and aOR 1.71 95% CI 1.62–1.80, respectively). In contrast, Glover et al. [40] (*n* = 1680 cases) examined the 20–39-year-old subset of the same database and evaluated alcohol abuse specifically (SNOMED-CT criteria) and found no statistically significant association (aOR 0.91, 95% CI 0.66–1.25).

In a US-based cross-sectional study of modest sample size (*n* = 156 cases), Zhang et al. [67] reported increased EOCRC risk among former drinkers (aOR 2.09 95% CI 1.01–4.36), but no significant association among current drinkers (aOR 1.23 95% CI 0.63–2.40).

Prospective cohort data suggested more consistent patterns, including evidence of dose–response relationships. In a nationwide Korean cohort (*n* = 8314 cases), Jin et al., 2023 [54] reported increased EOCRC risk among both moderate (10–20 g/day for women, 10–30 g/day for men; aHR 1.09, 95% CI 1.02–1.16) and heavy (≥20 g/day for women, ≥30 g/day for men; aHR 1.20, 95% CI 1.11–1.31) drinkers, with evidence of a dose–response (*p* < 0.0001) [54]. When stratified by sex, associations were only significant in men (moderate drinkers; aHR 1.09 95% CI 1.02–1.17, heavy drinkers; aHR 1.21 95% CI 1.11–1.31) [54]. Similarly, in the China Kadoorie Biobank study, Pan et al. [60] (*n* = 222 cases) observed increased risk among both former (aHR 3.54, 95% CI 1.10–11.35) and current (aHR 1.69, 95% CI 1.12–2.91) regular drinkers, with stronger associations in men (former regular: aHR 4.52 95% CI 1.38–14.79, current regular: aHR 1.94 95% CI 1.23–3.08).

Conversely, two smaller Canadian studies reported no statistically significant associations: Chang et al. [35] (*n* = 175 cases, aOR 1.07 95% CI 0.49–2.32) and O’Sullivan et al. [59] (*n* = 98 cases, daily use, aHR 1.72 95% CI 0.73–4.04).

#### 3.3.9. Smoking

Smoking was examined across twelve studies. Large registry-based analyses generally reported increased EOCRC risk amongst smokers. Danial et al. [37] (*n* = 13800 cases, aOR 1.59, 95% CI 1.54–1.65), Glover et al. [40] (*n* = 1680 cases, aOR 2.68, 95% CI 2.41–2.97) and Syed et al. [63] (*n* = 5710 cases, aOR 2.46, 95% 2.33–2.59) all reported increased EOCRC risk amongst ever-smokers. Elangovan et al. [66] (*n* = 16,090 cases) similarly observed increased risk among ever-smokers of all ages in men (ages 20–39: aOR 1.77, 95% CI 1.43–2.15; ages 40–49: aOR 1.25, 95% CI 1.16–1.36), and in women aged 40–49 years (aOR 1.16 95% CI 1.08–1.25) only, with non-significant findings among women aged 20–30 years (aOR 0.96, 95% CI 0.91–1.00).

In a cohort analysis, O’Sullivan et al. [59] (*n* = 98 cases) reported increased risk among current-heavy smokers (≥10 pack-years; aHR 1.87, 95% CI 1.00–3.52), whereas no significant associations were observed for light-smokers (aHR 1.13, 95% CI 0.25–5.16) or former-smokers (heavy-smoker aHR 1.21, 95% 0.56–2.63; light-smoker aHR 1.23 95% CI 0.72–2.11), noting that these estimates are based on HRs and reflect longitudinal cohort data. Puzzono et al. [44] reported a higher proportion of former-smokers among EOCRC cases compared with controls (29.8% vs. 1.4%, *p* < 0.0001), as well as a lower proportion of never-smokers (57.4% vs. 78.9%, *p* < 0.0001). The proportion of current smokers was also lower among cases than controls (12.8% vs. 19.7%, *p* < 0.0001); however, no adjusted effect estimates were provided.

Several smaller case–control and cross-sectional studies reported no significant association: Low et al. [41] (*n* = 651 cases, aOR 1.10, 95% CI 0.89–1.35), Zhang et al. [67] (*n* = 156 cases, aOR 0.79, 95% 0.47–1.33), Gausman et al. 2020 [38] (*n* = 269 cases, EOCRC 27% vs. controls 29%, *p* = 0.53), Pan et al. [60] (*n* = 222 cases; men aHR 1.69 95% CI 1.12–2.91; women aHR 1.45 95% CI 0.46–4.57), Chang et al. [35] (*n* = 175 cases, aOR 1.07 95% CI 0.68–1.67), and Collatuzzo et al. [36] (*n* = 189 cases, aOR 0.97 95% CI 0.57–1.65).

### 3.4. Comorbidities and Medications

#### 3.4.1. Diverticular Disease

Evidence regarding diverticular disease was limited, with only one US-based cohort study (Wang et al. [64]) evaluating this association, which reported a higher EOCRC risk (aOR 1.76, 95% CI 1.40–2.32).

#### 3.4.2. Medications

Four case–control studies evaluated medication as a primary exposure, including two large registry-based analyses (Nguyen et al. (*n* = 2557 cases) [43], Kane et al. (*n* = 1358 cases) [46]) and two smaller population-based studies (Chang et al. (*n* = 175 cases) [35], Low et al. (*n* = 651 cases) [41]). Antibiotic use was examined across three studies, with inconsistent findings and heterogeneity in both direction of association and effect measures. Nguyen et al. [43] reported no association with overall antibiotic use (aOR 1.06, 95% CI 0.96–1.17), although modestly increased risk was observed in adults with broad-spectrum antibiotic exposure (aOR 1.13, 95% CI 1.02–1.26). Similarly, Kane et al. [46] found no significant association across increasing categories of antibiotic exposure (stratified by no. of exposures; 1–3: 1.07 (95% CI 0.92–1.25), 4–6: 1.06 (95% CI 0.85–1.32), 7–9: 1.07 (95% CI 0.80–1.44), >9: 1.15 (95% CI 0.87–1.54)). Chang et al. [35], however, reported reduced EOCRC risk with childhood antibiotic exposure (<20 years) (aOR 0.29, 95% CI 0.09–0.90), while no association was observed for adult use (aOR 0.78, 95% CI 0.47–1.30).

Aspirin use demonstrated similarly mixed findings. Low et al. [41] (aOR 0.66, 95% CI 0.52–0.84) reported a significant protective effect of aspirin, whereas Chang et al. [35] reported no significant association (aOR 1.20, 95% CI 0.75–1.92).

### 3.5. Early Life Factors

Gausman et al. [39] conducted a single-centre case–control study (*n* = 269 cases) and reported no significant association between EOCRC risk and early life exposures, including breastfeeding (aOR 1.04, 95% CI 0.84–1.29), maternal smoking (aOR 0.94, 95% CI 0.77–1.15), childhood obesity (aOR 1.04, 95% CI 0.85–1.26) or time of puberty (menarche for females (≤11 vs. ≥14 aOR 1.19, 95% CI 0.85–1.68); first facial hair for males aOR 0.64, 95% CI 0.38–1.10).

Cao et al. [34], in a nationwide case–control study (*n* = 564 cases), observed a positive association between birth by caesarean section and EOCRC risk among females only (aOR 1.62, 95% CI 1.01–2.60).

## 4. Discussion

The rising incidence of sporadic EOCRC urgently demands a better understanding of disease aetiology. While interpretation of incidence trends should consider advances in diagnostic technologies such as colonoscopy, increased clinical awareness and improvements in cancer registry ascertainment, these factors are unlikely to explain the age-specific divergence currently observed, whereby EOCRC rates are increasing while average-onset disease remains stable or declining in many settings. Instead, the rise appears to coincide with multiple features of modern, industrialised societies, including dietary patterns, sedentary lifestyles, and metabolic risk factors, which were consistently identified in this review and often co-occur, making their individual contributions to risk difficult to disentangle [14,71,72,73,74]. Several hypothesised risk exposures, including microplastics, have biological plausibility supported by pre-clinical models [75] and observational tissue studies [76], but have not yet been evaluated in epidemiologic studies and were therefore not included in this review.

In this systematic review, the most robust evidence, derived primarily from large registry-based studies, supported associations between EOCRC risk and family history, as well as metabolic factors including obesity. Notably, associations with family history persisted even in studies excluding recognised hereditary cancer syndromes, suggesting additional inherited susceptibility, although shared familial or environmental exposures may also contribute.

### 4.1. Non-Modifiable Risk Factors

Family history was among the most consistent predictors of EOCRC, even after excluding individuals with known hereditary cancer syndromes or inflammatory bowel disease [38,40]. This pattern may reflect underlying susceptibility that is not fully captured or characterised by current genetic classifications, as well as the potential contribution of gene–environment interactions related to diet, lifestyle or socioeconomic factors. These findings further support the patterns of familial clustering observed in EOCRC, consistent with the conclusions of Hua et al. [25] and Ye et al. [27]. However, as genetic status was not directly evaluated in these studies, and classification of hereditary risk was largely based on reported family history or registry data rather than systematic germline testing, these observations may also reflect shared familial or environmental exposures and their interplay.

Beyond identifiable genetic syndromes, other potentially heritable biological traits may contribute to EOCRC risk. Patterns of cellular ageing and genomic instability, which are at least partly heritable and are established contributors to average-onset colorectal cancer, may also be relevant in younger patients. Telomere shortening has been proposed as a potential biomarker of early carcinogenesis. Martel et al. [42] and Qin et al. [77] reported shorter telomere length in individuals with EOCRC or lower baseline risk, whereas other studies have found no significant association [78,79].

Polygenic risk scores (PRS) are one way of capturing complex genetic susceptibility across large populations. PRS have been explored in colorectal cancer as a means of improving individual risk prediction [80,81,82,83]. Notably, outside the predefined search window of our review, Sandhu et al. [84] utilised the UK Biobank to conduct a case–control study across 31,164 matched individuals for factors including markers of biological (rather than chronological) age and PRS for CRC (derived from PMID: 31866242). Greater excess of biological versus chronological age was associated with greater EOCRC risk, while higher CRC PRS portended higher risk of CRC, particularly EOCRC (OR 1.53; 95% CI 1.19–1.97; *p* < 0.001). Collectively, these findings support a model in which EOCRC arises from the interaction between inherited susceptibility and environmental exposures acting across a lifetime. This is clinically relevant, as current screening guidelines prioritise early screening for those with a strong family history [85,86], not utilising the complementary capacity of PRS to better select at-risk individuals.

Demographic factors, including age [41,67], male sex [37,38,40,41,63] and Caucasian ethnicity [40,63,67], were also associated with increased EOCRC risk. Most diagnoses occurred between the ages of 40 and 49 years, confirming that increasing cancer risk with age applies even within ‘young’ cohorts. Although cases in individuals ≥45 may now be captured by recent shifts in screening recommendations, a substantial proportion of EOCRC cases remain outside screening-eligible populations [87]. These conclusions are largely drawn from US-based studies and should be interpreted in the context of socioeconomic disparities impacting screening uptake, healthcare access and data reporting in US healthcare.

Future research should integrate demographic, familial, and polygenic risk information to refine early identification strategies, particularly in populations currently classified as ‘average-risk’.

### 4.2. Modifiable Risk Factors

Among modifiable exposures, metabolic dysfunction and obesity were most consistently associated with EOCRC risk across several large registry-based studies, despite differing effect measures. Both childhood and adult obesity were implicated in the majority of studies [37,40,45,48,52,53,57,60,61,62,63,66] and across geographical regions, with a dose–response relationship observed in some [53,57], with only smaller institutional studies reporting null findings [38,59,67]. These associations may be mediated by chronic low-grade inflammation, hormonal dysregulation and gut microbiome disruption, which may promote a pro-tumorigenic microenvironment through altered immune signalling, increased cellular proliferation, and impaired apoptosis within the colorectal epithelium [88,89,90]. Hua et al. similarly implicated the global rise in obesity as a key driver of incidence trends [25]. Notably, two studies reported inverse associations [35,41], which may reflect unintentional weight loss preceding diagnosis of EOCRC.

While isolated metabolic conditions, including hypertension, diabetes and dyslipidaemia, have shown variable contribution to EOCRC risk, their co-occurrence demonstrated consistent associations [37,40,45,47,48,50,52,53,60,61,63,66]. This clustering of risk factors suggests a broader state of metabolic dysfunction, which may contribute to pathways involved in colorectal carcinogenesis through insulin resistance, oxidative stress, hormonal and microbiome disruption, with hyperinsulinaemia and associated signalling pathways further promoting tumour growth and progression [47,88,91,92,93,94].

Aligning with this, sedentary behaviours, known to contribute to abdominal adiposity and insulin resistance, were also associated with EOCRC in two large population-based studies, with some evidence suggesting a stronger association with rectal cancer [35,58], reinforcing results seen in recent reviews [25,27]. Interestingly, the left-sided predominance observed in EOCRC parallels the distribution seen in colitis, suggesting potential shared lifestyle exposures that may influence the gut microbiome and manifestation of disease [73,95,96,97], potentially through region-specific alterations in microbial composition and local inflammatory responses. Higher levels of physical activity were not consistently found to reduce EOCRC risk, although these findings came from smaller studies of lower evidence levels. This also highlights the need for better characterisation of dose, timing, duration, and domain of activity exposure [44,59,67].

Behavioural patterns broadly converge with dietary patterns, with the most reproducible signals relating to overall diet quality rather than individual foods. Diets characterised by high intake of meat, processed foods and added sugars alongside low fibre, often termed ‘Western dietary patterns’, as well as hyperinsulinaemic diets and high consumption of sugar-sweetened beverages, were consistently associated with EOCRC risk [35,36,44,51,65]. In contrast, evidence for individual food groups, including red and processed meats, was heterogeneous. Notably, Pan et al. [60] reported higher EOCRC risk with frequent fish consumption, but not red meat, in a Chinese cohort. This finding may reflect a regional dietary pattern in which red meat intake is comparatively low and may not reach risk-associated thresholds. Data linking fish consumption to EOCRC risk remains limited; however, some studies have hypothesised that environmental pollutants in rapidly industrialising regions, such as pesticides and microplastics contaminating aquatic ecosystems, may contribute to carcinogenesis through disruption of the microbiome rather than through the biological effect of fish itself [71,72]. Evidence for a protective effect of fruit and vegetable intake was also inconsistent [35,59]. Our review expands on observations seen in Hua et al. and Ye et al. [25,27], by emphasising the broader concept of poor diet quality, particularly hyperinsulinaemic diets [35,65], which may be associated with pathways involved in carcinogenesis through metabolic and inflammatory pathways. Proposed mechanisms include the formation of N-nitroso compounds, increased reactive oxygen species, colonic inflammation, and gut dysbiosis [98,99,100], all of which may contribute to DNA damage, epithelial barrier dysfunction, and early neoplastic transformation.

Vitamin D intake was examined across three studies (*n* = 529 cases) and was consistently linked to reduced EOCRC risk [35,59], suggesting a potential protective association [36,55,56]. While sunlight exposure was not measured, effects were independent of geographical location and physical activity. These findings raise the possibility that vitamin D may represent a modifiable risk factor in EOCRC risk, although causality cannot be inferred, and further studies are required to determine whether dietary optimisation or supplementation could play a role in prevention strategies.

By contrast, while alcohol and tobacco are well-established carcinogens, their associations with EOCRC were less consistent [25,27] in our review. Smoking was associated with increased EOCRC risk in several large registry-based analyses [37,40,63,66], although temporal trends were not consistent [44,59]. Some studies suggested that earlier initiation or prior exposure may confer cumulative risk beyond current smoking status [35,37,40,54,59,60,63,67], while several smaller cohorts reported no significant association [36,38,41,60,67]. Evidence regarding alcohol exposure was similarly heterogeneous, with variability likely reflecting differences in drinking patterns, population demographics, definitions of alcohol exposure, and interactions with genetic and lifestyle factors that modulate alcohol-related carcinogenesis [21,101,102].

The role of pharmacological agents in EOCRC remains insufficiently characterised, with recent reviews also noting a paucity of high-quality EOCRC-specific data [25,27].

Overall, antibiotic exposure was not associated with EOCRC risk in two large-scale studies [35,43,46], contrasting with studies in later-onset CRC that have reported modest but consistent associations [103,104,105]. However, modest increases in risk were observed with certain patterns of adult antibiotic exposure [43], while one study reported an inverse association with childhood exposure [35]. These findings highlight ongoing uncertainty regarding timing, duration and spectrum of antibiotic exposure most relevant to colorectal carcinogenesis. They may also suggest that microbiome disruption occurring in adulthood may differ from that in early life, or that chronic dysbiosis plays a more prominent role in later-onset CRC than EOCRC. More broadly, the mechanisms linking microbiome perturbations with cancer risk remain poorly defined [14,97,106,107].

Overall, evidence was strongest and most consistent for metabolic and lifestyle-related factors, including obesity and metabolic dysfunction, whereas findings for dietary components, environmental exposures, and pharmacological factors were more heterogeneous and limited, reflecting variability in exposure measurement and study design. Given the observational and predominantly retrospective nature of the included studies, all findings were interpreted as associations rather than evidence of causality and may reflect residual confounding or unmeasured biases.

## 5. Limitations

This systematic review has several methodological considerations. Although the search strategy was developed in consultation with an academic librarian and conducted in accordance with PRISMA guidelines, it was designed to prioritise sensitivity using broad exposure-related terms rather than exhaustive exposure-specific keywords. This may have resulted in the omission of studies not indexed using general risk factor terminology. Additionally, the search was limited to two databases and English-language publications, which may have resulted in the omission of relevant studies.

A meta-analysis was considered but not undertaken due to substantial heterogeneity in exposure definitions, outcome ascertainment, covariate adjustment models and reporting of effect measures across studies. Consistent with SWiM guidance [33], synthesis therefore relied on structured thematic methods, including grouping of studies and direction-of-effect assessment. While this approach allows transparent synthesis of heterogeneous evidence, it does not generate pooled effect estimates and involves some degree of interpretative judgement. In synthesising the evidence, greater weight was given to larger prospective studies and those incorporating robust confounder adjustment, and all included studies met a predefined threshold for methodological quality (NOS ≥ 6). While this approach ensured a minimum standard of study quality, it may have excluded lower-quality studies and does not fully eliminate heterogeneity in study design, exposure assessment, and residual confounding. More broadly, the included studies were subject to several domain-specific limitations, including potential selection bias (particularly in registry-based and case–control designs), recall bias in self-reported exposures and exposure misclassification. Surveillance and detection bias may also have influenced observed associations, particularly in populations with differing access to healthcare and screening practices.

Additionally, the definition of ‘sporadic’ EOCRC across included studies was variable and often based on exclusion of recognised hereditary syndromes rather than comprehensive germline testing, which may have resulted in misclassification of cases with unrecognised genetic predisposition.

Finally, the predominance of data from high-income Western countries may limit generalisability to other populations.

Despite these limitations, this review synthesises a large heterogeneous body of evidence examining risk factors for sporadic EOCRC. The review was prospectively registered through PROSPERO, applied predefined eligibility criteria, incorporated dual-reviewer screening and quality assessment, and provides an updated synthesis of a rapidly evolving evidence base, including studies not captured in prior reviews.

## 6. Conclusions

This systematic review highlights consistent associations between sporadic EOCRC and several modifiable risk factors, including Western dietary patterns, metabolic dysfunction and physical inactivity. Non-modifiable factors, including male sex, Caucasian ethnicity and family history of colorectal cancer, were also implicated, with clear signals of heritable risk beyond known cancer syndromes. These findings were interpreted as associative rather than causal and serve to generate hypotheses for future mechanistic and prospective studies. Future research should aim to clarify the independent and interacting contributions of these factors, including the role of polygenic susceptibility, to inform targeted prevention strategies for EOCRC.

## Figures and Tables

**Table 1:** Study characteristics.

	First Author (Year) (Reference)	Location	Population Selection	Study Design (Period)	Quality Scores	Age of EOCRC Cohort (Mean/Median at Diagnosis)	Total Sample Size	Sample Size (Cases)	Sex (% Male)	Risk Factors Evaluated	Key Finding (Direction of Association) *
Case–Control Studies											
1	Cao (2023) [34]	Sweden	Epidemiology Strengthened by Histopathology Reports in Sweden (ESPRESSO) cohort	Retrospective Case–Control (1991–2017)	9	18–49 (mean 32.9)	2744	564	50	Birth by caesarean section vs. by vaginal delivery	**Positive associations:** caesarean section in females only.
2	Chang (2021) [35]	Canada	Ontario Cancer Registry (OCR)	Retrospective Case–Control (2018–2019)	6	20–49 (mean 43.1)	428	175	42	Family History, Diet, Weight-related metrics, Comorbidities, Medications, Parity, OCP use, Menopausal status, Smoking, Alcohol	**Positive associations:** FHx of CRC, SSB intake, and sedentary behaviours. **Inverse associations:** Parity, ABx use in <20 yo only. **No significant associations:** processed meat intake, red meat intake, vegetable and fruit intake, BMI categories, alcohol and smoking, T2DM.
3	Collatuzzo (2024) [36]	Iran	IROPICAN study	Retrospective Case–Control (2017–2020)	6	18–49 (mean not reported)	378	189	51	Family history, Diet, Weight-related metrics, Smoking, Physical activity, Medications, Socioeconomic status	**Positive associations:** FHx of CRC, and red meat intake (>35.6 g/day). **Inverse associations:** vegetable intake (422–576 g/day). **No significant associations:** fruit intake, vegetable intake (>576 g/day), red meat intake (12.83–25.64 g/day), smoking.
4	Danial (2022) [37]	USA	IBM Watson Health Explorys Dataset	Retrospective Case–Control (1999–2019)	8	20–50 (mean not reported)	1,371,115	13,800	49	Gender, Weight-related metrics, Family History, Smoking, Alcohol	**Positive associations:** FHx of CRC, male sex, obesity/metabolic comorbidity, smoking and alcohol use.
5	Gausman (2020) [38]	USA	NYU Langone Health Academic Medical Centre	Retrospective Case–Control (2011–2017)	8	18–49 (mean 43.0)	1391	269	54	Gender, Family History, Weight-related metrics, Comorbidities, Smoking	**Positive associations:** FHx of CRC, male sex. **Inverse associations:** hyperlipidaemia. **No significant associations:** BMI, Hypertension, diabetes, and smoking.
6	Gausman (2022) [39]	UK	UK Biobank cohort participants	Prospective Case–Control (2006–2019)	8	38–49 (mean not reported)	451,615	455 prevalent cases 85 incident cases	51	Early life factors	**No significant associations.** Early life factors evaluated included: breastfeeding, childhood body size, growth patterns and puberty timing.
7	Glover (2019) [40]	USA	IBM Watson Health Explorys Dataset	Retrospective Case–Control (2013–2018)	9	20–39 (mean not reported)	8,873,080	1680	48	Gender, Race, Family History, Weight-related metrics, Comorbidities, Smoking, Alcohol	**Positive associations:** FHx of non-CRC, Caucasian ethnicity, diabetes, smoking. **Inverse associations:** male sex. **No significant associations:** alcohol.
8	Low (2020) [41]	USA	Veterans’ Health Administration databases	Retrospective Case–control (1999–2014)	7	18–49 (mean 44.8)	68,067	651	91	Age, Gender, Weight-related metrics, Medications, Smoking	**Positive associations:** increasing age and male sex. **Inverse associations:** obesity, aspirin use. **No significant associations:** underweight.
9	Martel-Martel (2023) [42]	Spain	Multi-centre study (Salamanca, Barcelona, Madrid, Leon, Vizcaya)	Prospective Case–control (2022)	7	18–49 (mean not reported)	196	87 (70 for genetic analysis)	Not reported	Telomere length in leukocytes, Genetic variation in telomere maintenance genes	**Inverse associations:** absolute leukocyte telomere length shorter in EOCRC cases vs. controls; 122 kb vs. 296 kg, *p* < 0.001
10	Nguyen (2022) [43]	Sweden	Epidemiology Strengthened by histopathology Reports in Sweden (ESPRESSO) cohort	Retrospective Case–control (2006–2016)	9	18–49 (mean 42.9)	15,197	2557	54	Antibiotics	**Positive associations:** broad-spectrum antibiotic use. **No significant associations:** overall antibiotic use, narrow-spectrum/anti-aerobic/anti-anaerobic antibiotic use.
11	Puzzono (2021) [44]	Italy	Milan, third-level academic hospital	Retrospective Case–control (2018–2020)	7	18–49 (mean not reported)	118	47	55	Family history, Diet, Physical activity, Smoking	**Positive associations:** FHx of CRC, fresh meat intake, processed meat intake. **No significant associations:** physical activity.
**Nested Case–Control Studies**											
1	Chen (2021) [45]	USA	IBM MarketScan Commercial Database	Retrospective Case–Control (2006–2015)	9	18–49 (mean 43.0)	23,365	4673	52	Metabolic syndrome and conditions	**Positive associations:** metabolic syndrome and increasing number of metabolic comorbidities.
2	Kane (2025) [46]	USA	Kaiser Permanente Northern California cohort	Retrospective Case–control (1998–2020)	9	18–49 (mean not reported)	6070	1359	53	Medications	**No significant associations:** antibiotic use and EOCRC risk across all antibiotic subtypes.
3	Li (2022) [47]	USA	IBM MarketScan Commercial Database	Retrospective Case–Control (2006–2015)	8	18–49 (mean 43.0)	58,105	6001	51	Diabetes	**Positive associations:** T2DM overall (increased with uncontrolled or complicated disease). **No significant associations:** controlled T2DM.
4	Lundqvist (2023) [48]	Sweden	Colorectal Cancer Database (CRCBaSe) linked to Swedish Colorectal Cancer Registry	Retrospective Case–Control (2007–2016)	8	18–49 (mean 42.5)	18,382	2626	54	Metabolic conditions, Comorbidities	**Positive associations:** metabolic disease (stronger association in individuals with co-existing IBD).
**Cohort Studies**											
1	Agazzi (2020) [49]	Italy	Operative Unit of Digestive Endoscopy, Fondazione IRCCS Policlinico San Matteo, University of Pavia	Retrospective Cohort (2015–2018)	7	18–49 (mean 42.5)	1778	27	59	Gender, Family History	**No significant associations:** FHx of CRC, sex.
2	Chang (2024) [50]	South Korea	National Healthcare Insurance Service (NHIS)	Retrospective Cohort (2009–2011)	9	20–49 (mean not reported)	3,340,635	7492	64	Hypertriglyceridaemia	**Positive associations:** hypertriglyceridaemia (including persistent hypertriglyceridaemia)
3	Hur (2021) [51]	USA	Nurses’ Health Study II (NHS II)	Prospective Cohort (1991–2015 recruitment)	8	25–42 at enrolment (mean not reported)	95,464	109	0	Diet	**Positive associations:** higher SSB intake. **No significant associations:** low-to-moderate SSB intake.
4	Jimba (2021) [52]	Japan	JMDC Claims Database	Retrospective Cohort (2005–2018)	8	20–49 (mean not reported)	902,599	1884	55	Metabolic Syndrome	**Positive associations:** metabolic syndrome overall, and metabolic syndrome among males. **No consistent associations**: female sex.
5	Jin (2022) [53]	South Korea	National Healthcare Insurance Service (NHIS)	Retrospective Cohort (2009–2010, follow up to 2019)	8	20–49 (median 46)	5,672,153	8320	56 (non-MetS group), 80 (MetS group)	Weight-related metrics, Metabolic Syndrome, Comorbidities	**Positive associations:** metabolic syndrome, obesity, central adiposity, fasting glucose, hypertriglyceridaemia.
6	Jin (2023) [54]	South Korea	National Healthcare Insurance Service (NHIS)	Retrospective Cohort (2009–2019)	8	20–49 (mean not reported)	5,666,576	8314	59	Alcohol	**Positive associations:** moderate-to-heavy alcohol consumption (only significant in males). **No significant associations:** non-drinkers.
7	Kim (2021) [55]	USA	Nurses’ Health Study II (NHS II)	Prospective Cohort (1991–2015 recruitment)	7	25–42 at enrolment (mean not reported)	94,205	111	0	Vitamin D	**Inverse associations:** higher total and dietary vitamin D intake. **No significant associations:** vitamin D supplement intake.
8	Kim (2023) [56]	South Korea	Kangbuk Samsung Health Study	Retrospective Cohort (2011–2018)	8	18–49 (median 42.4 amongst incident cases)	212,885	229	Reported by vitamin D level (<10 ng/mL: 32, 10–19 ng/mL: 55, ≥20 ng/mL: 66)	Vitamin D	**Inverse associations:** higher circulating vitamin D levels.
9	Liu (2019) [57]	USA	Nurses’ Health Study II (NHS II)	Prospective Cohort (1989–2011 recruitment)	8	25–42 at enrolment (median 45)	85,256	114	0	Weight-related metrics	**Positive associations:** obesity and weight gain during adulthood. **No significant associations:** BMI in early adulthood.
10	Nguyen (2018) [58]	USA	Nurses’ Health Study II (NHS II)	Prospective Cohort (1991–2011 recruitment)	8	25–42 (median 45)	89,278	118	0	Sedentary Behaviours	**Positive associations:** prolonged sedentary time (high TV viewing time) (14 h/week). **No significant associations:** moderate sedentary time (7.1–14 h/week).
11	O’Sullivan (2024) [59]	Canada	Ontario Health Study (OHS) and Alberta’s Tomorrow Project (ATP) cohorts	Prospective Cohort (OHS 2009–2017, ATP 2000–2015)	9	18–49 (mean not reported)	127,852	98	35 (OHS), 35 (ATP)	Gender, Family History, Socioeconomic factors, Diet, Weight-related metrics, Comorbidities, Smoking, Alcohol, Parity, OCP use	**Positive associations:** FHx of CRC, current heavy smoking. **No significant associations:** sex, BMI, waist circumference, physical activity, diabetes, hypertension, diet, alcohol, parity and smoking overall.
12	Pan (2023) [60]	China	China Kadoorie Biobank (CKB) participants	Prospective Cohort (2004–2008 recruitment)	7	30–50 (median 46.3)	88,055	222	38	Diet, Weight-related metrics, Comorbidities, Smoking, Alcohol	**Positive associations:** FHx of any cancer (significant among males), fish intake, diabetes, hypertension, BMI (overall), smoking and alcohol use. **No significant associations:** Red meat, poultry and egg intake.
13	Park (2023) [61]	South Korea	National Health Insurance Service (NHIS)	Retrospective Cohort (2009–2012)	9	20–39 (mean not reported)	5265,590	7910	57	Metabolic conditions	**Positive associations:** NAFLD
14	Song (2023) [62]	South Korea	National Health Insurance Service (NHIS)	Retrospective Cohort (2009–2011)	8	20–49 (mean not reported)	7,710,534	7492	63	Weight-related metrics	**Positive associations:** obesity and abdominal adiposity (particularly when persistent across repeated measurements).
15	Syed (2019) [63]	USA	IBM Watson Health Explorys Dataset	Retrospective Cohort (2012–2016)	7	24–49 (mean not reported)	11,806,130	5710	49	Gender, Race, Family History, Weight-related metrics, Comorbidities, Smoking, Alcohol	**Positive associations:** FHx of any cancer, FHx of GI cancer, male sex, Caucasian ethnicity (vs. non-Caucasian), obesity, hypertension, hyperlipidaemia, smoking and alcohol use. **No significant associations:** FHx of polyps.
16	Wang (2022) [64]	USA	TriNetX analytics network platform	Retrospective Cohort (2010–2021)	7	20–49 (mean not reported)	46,179,351	571 (with diverticular disease), 300 (without diverticular disease)	52	Diverticular Disease	**Positive associations:** diverticular disease.
17	Yue (2021) [65]	USA	Nurses’ Health Study II (NHS II)	Prospective Cohort (1981–2015)	8	25–42 (mean 45)	94,217	111	0	Diet	**Positive associations:** hyperinsulinaemic lifestyle index. **No significant associations:** prime diet quality score, plant-based diet index, empirical dietary index for hyperinsulinaemia.
**Cross-Sectional Studies**											
1	Elangovan (2021) [66]	USA	IBM Watson Health Explorys Dataset	Cross-Sectional (2017–2021)	7	20–50 (mean not reported)	37,483,140	16,090	16	Weight-related metrics, Comorbidities, Smoking	**Positive associations:** Obesity, diabetes (among males), hypertension, hyperlipidaemia and smoking (predominantly in male and older cohorts). **No significant associations:** diabetes (among females), hypertension (among 20–39 yo females).
2	Zhang (2024) [67]	USA	National Health Interview Survey (NHIS USA)	Cross-sectional (2004–2018)	6	18–49 (mean 41.8)	205,002	156	54	Age, Gender, Race, Weight-related metrics, Comorbidities, Smoking, Alcohol	**Positive associations:** increasing age, and former alcohol use. **Inverse associations:** Hispanic ethnicity (vs. non-Hispanic white), moderate-to-vigorous physical activity. **No significant associations:** female sex, obesity, smoking and light/moderate current alcohol use.

* Effect estimates (crude or adjusted OR/HR/RR) varied across studies and therefore are not directly comparable. This table summarises the direction of associations only. Full effect estimates and exposure definitions are provided in Supplementary Materials Table S1.

**Table 2:** Metabolic syndrome definitions.

Study	Definition of Metabolic Syndrome
Jin et al., 2022 [53]	≥3 of: Abdominal obesity (≥90 cm in men, ≥80 cm in women) Triglycerides ≥ 150 mg/dL, HDL-C (<40 mg/dL in men, <50 mg/dL in women) BP ≥ 130/85 mmHg or anti-hypertensive use Fasting glucose ≥ 100 mg/dL or use of antidiabetic medications
Jimba et al. [52]	Abdominal obesity (≥95 cm in men and ≥85 cm in women) AND ≥2 of: BP ≥ 130/85 mmHg or antihypertensive use Triglycerides ≥ 150 mg/dL or HDL-C (<40 mg/dL) or use of lipid-lowering medication Fasting glucose ≥ 110 mg/dL or use of antidiabetic medication
Chen et al., 2021 [45]	ICD-9-CM diagnostic code (277.7) for metabolic syndrome OR at least ≥3 of: (also as defined by ICD-9-CM codes) Obesity/Overweight Hypertension or antihypertensive use Hyperlipidaemia or lipid-lowering medication use Hyperglycaemia or type 2 diabetes mellitus or anti-diabetic medication use
Lundqvist et al. [48]	Metabolic disease defined as ≥1 of: (ICD-10-CM codes and Prescribed Drug register) T2DM or use of antidiabetic medications Hypertension or use of antihypertensives Disorders of lipoprotein metabolism, dyslipidaemia or use of lipid-lowering medication Fatty change of liver *Metabolic syndrome is not specifically defined; however, the authors investigated the risk based on no. of metabolic diseases.*

## Data Availability

No new data were created in this study. The data supporting the findings of this systematic review are included within the article and its Supplementary Materials.

## Supplementary Materials

The following supporting information can be downloaded at: https://www.mdpi.com/article/10.3390/cancers18101515/s1, Table S1: Study Quality Assessments; Table S2: Primary Study Findings; PRISMA Abstract Checklist; PRISMA Manuscript Checklist.
