# Supplementary material for: Risk Factors in Sporadic Early-Onset Colorectal Cancer, Current Evidence and Emerging Insights: A Systematic Review
Source: Cancers (Basel). 2026 May 8;18(10):1515. doi: 10.3390/cancers18101515 (PMC13204181; doi:10.3390/cancers18101515)
Supplement: Supplementary file 1 [file cancers-18-01515-s001.zip › cancers-4285736-supplementary.pdf]

SUPPLEMENTARY MATERIALS

PUBMED SEARCH STRATEGY

Search number	Query
9	((("Colorectal Neoplasms"[MeSH Terms:noexp] OR "Colonic Neoplasms"[MeSH Terms] OR "Rectal Neoplasms"[MeSH Terms]) OR ("colon tumor"[Title/Abstract] OR "colon malign*"[Title/Abstract] OR "colon canc*"[Title/Abstract] OR "rectum tumor"[Title/Abstract] OR "rectum malign*"[Title/Abstract] OR "rectum canc*"[Title/Abstract] OR "colorectal tumor"[Title/Abstract] OR "colorectal malign*"[Title/Abstract] OR "colorectal canc*"[Title/Abstract] OR "CRC"[Title/Abstract])) AND ("young-onset"[Title/Abstract] OR "early-onset"[Title/Abstract] OR "under 50"[Title/Abstract:~3] OR "younger 50"[Title/Abstract:~3] OR "young-onset"[Title/Abstract] OR "early-onset"[Title/Abstract])) AND (("Risk Factors"[MeSH Terms] OR "Comorbidity"[MeSH Terms] OR "Life Style"[MeSH Terms]) OR (("risk factors"[Title/Abstract] OR "risk*"[Title/Abstract] OR "factor*"[Title/Abstract]).kf))
8	((("Colorectal Neoplasms"[MeSH Terms:noexp] OR "Colonic Neoplasms"[MeSH Terms] OR "Rectal Neoplasms"[MeSH Terms]) OR ("colon tumor"[Title/Abstract] OR "colon malign*"[Title/Abstract] OR "colon canc*"[Title/Abstract] OR "rectum tumor"[Title/Abstract] OR "rectum malign*"[Title/Abstract] OR "rectum canc*"[Title/Abstract] OR "colorectal tumor"[Title/Abstract] OR "colorectal malign*"[Title/Abstract] OR "colorectal canc*"[Title/Abstract] OR "CRC"[Title/Abstract])) AND ("young-onset"[Title/Abstract] OR "early-onset"[Title/Abstract] OR "under 50"[Title/Abstract:~3] OR "younger 50"[Title/Abstract:~3] OR "young-onset"[Title/Abstract] OR "early-onset"[Title/Abstract])) AND (("Risk Factors"[MeSH Terms] OR "Comorbidity"[MeSH Terms] OR "Life Style"[MeSH Terms]) OR (("risk factors"[Title/Abstract] OR "risk*"[Title/Abstract] OR "factor*"[Title/Abstract]).kf))
7	("Risk Factors"[MeSH Terms] OR "Comorbidity"[MeSH Terms] OR "Life Style"[MeSH Terms]) OR (("risk factors"[Title/Abstract] OR "risk*"[Title/Abstract] OR "factor*"[Title/Abstract]).kf)
6	("Colorectal Neoplasms"[MeSH Terms:noexp] OR "Colonic Neoplasms"[MeSH Terms] OR "Rectal Neoplasms"[MeSH Terms]) OR ("colon tumor"[Title/Abstract] OR "colon malign*"[Title/Abstract] OR "colon canc*"[Title/Abstract] OR "rectum tumor"[Title/Abstract] OR "rectum malign*"[Title/Abstract] OR "rectum canc*"[Title/Abstract] OR "colorectal tumor"[Title/Abstract] OR "colorectal malign*"[Title/Abstract] OR "colorectal canc*"[Title/Abstract] OR "CRC"[Title/Abstract])
5	("risk factors"[Title/Abstract] OR "risk*"[Title/Abstract] OR "factor*"[Title/Abstract]).kf
4	"Risk Factors"[MeSH Terms] OR "Comorbidity"[MeSH Terms] OR "Life Style"[MeSH Terms]
3	"young-onset"[Title/Abstract] OR "early-onset"[Title/Abstract] OR "under 50"[Title/Abstract:~3] OR "younger 50"[Title/Abstract:~3] OR "young-onset"[Title/Abstract] OR "early-onset"[Title/Abstract]
2	"colon tumor"[Title/Abstract] OR "colon malign*"[Title/Abstract] OR "colon canc*"[Title/Abstract] OR "rectum tumor"[Title/Abstract] OR "rectum malign*"[Title/Abstract] OR "rectum canc*"[Title/Abstract] OR "colorectal tumor"[Title/Abstract] OR "colorectal malign*"[Title/Abstract] OR "colorectal canc*"[Title/Abstract] OR "CRC"[Title/Abstract]
1	"Colorectal Neoplasms"[MeSH Terms:noexp] OR "Colonic Neoplasms"[MeSH Terms] OR "Rectal Neoplasms"[MeSH Terms]

EMBASE SEARCH STRATEGY

Search number	Query
1.	exp colorectal cancer/
2.	("colon tumo?r" or "colon malign*" or "colon canc*" or "rect* tumo?r" or "rect* malign*" or "rect* canc*" or "colorectal tumo?r" or "colorectal malign*" or "colorectal* canc*" or CRC).ti,ab.
3.	1 or 2
4.	("young onset" or "early onset" or "young-onset" or "early-onset" or "under 50" or "under the age of 50" or "younger than 50" or "under adj4 50").ti,ab.
5.	exp risk factor/
6.	exp comorbidity/
7.	exp lifestyle/
8.	("risk factor" or "risk*" or "factor*").tw,kf.
9.	5 or 6 or 7 or 8
10.	3 and 4 and 9
11.	limit 10 to english language

ELIGIBILITY CRITERIA

Inclusion:

1. Published in full-text format
2. Study design
    a. Original study using epidemiologic design (either prospective or retrospective) e.g. cohort, case control, and cross-sectional design
3. Quality
    a. Published in peer-reviewed journal
4. Population
    a. Patient population with CRC and focused on CRC in <50 years old (18-49 years old). If studies included patients of all ages, the data had to be reported separately by age, with an upper limit for the age comparison group of 50 years.
    b. Adenocarcinoma histology
5. Study themes
    a. Focused on sporadic CRC
    b. Discussed at least one of the following areas
        i. Risk factors – environmental, lifestyle, genome, microbiome
        ii. Lifestyle and environmental factors – smoking, alcohol consumption, activity level, occupational exposures
        iii. Clinical/comorbidities – obesity, T2DM, HTN etc.
        iv. Genomic and Genetic factors

Exclusion

1. Articles discussing hereditary CRC syndromes only
2. Grey-literature (e.g. annual reports from cancer societies)
3. Reviews and meta-analyses were also excluded, but their bibliographies were searched (include as separate group for now)
    a. If including them in Umbrella review, or if including, need to ensure checking reference lists to ensure no duplication of references (can only include if conducting an umbrella review
    b. “We conducted backward hand searching for all systematic reviews identified in the database searches and hand searched reference lists of included articles”
4. Meeting abstracts, reviews, non-English articles, and non-original research were excluded.

5. Excluded studies that had different age cut off to 50 years old
6. Excluded studies that compared to LOCRC, rather than independently looking at cohort of <50 years old or comparing with healthy controls*

*This decision was made, as we recognise that risk factors may not be mutually exclusive between EOCRC and LOCRC. Therefore, we planned to include studies that either independently assessed EOCRC or compared to individuals without cancer.

TABLE S1. STUDY QUALITY ASSESSMENTS.

Case-Control Studies									
	Case	Representative	Controls	Definition	Comparability/ Confounding (2)	Exposure	Method	Non-response	Score
Cao 2023[34]	*	*	*	*	**	*	*	*	9
Chang 2021[35]	*	*	*	*	*	-	-	*	6
Collatuzzo 2024[36]	*	*	*	*	*	*	-	-	6
Danial 2022[37]	*	*	*	*	*	*	*	*	8
Gausman 2020[38]	*	*	-	*	**	*	*	*	8
Gausman 2021[39]	*	*	*	*	**	*	-	*	8
Glover 2019[40]	*	*	*	*	**	*	*	*	9
Low 2020[41]	*	-	*	*	*	*	*	*	7
Martel-Martel 2023[42]	*	*	*	*	*	*	*	-	7
Nguyen 2022[43]	*	*	*	*	**	*	*	*	9
Puzzono 2022[44]	*	*	*	*	*	*	*	-	7
Nested Case-Control Studies									
Chen 2021[45]	*	*	*	*	**	*	*	*	9
Kane 2024[46]	*	*	*	*	**	*	*	*	9

Li 2022[47]	-	*	*	*	**	*	*	*	8
Lundqvist 2023[48]	*	*	*	*	*	*	*	*	8

Cohort Studies									
	Representative	Non-exposed	Exposure	Outcome not present	Confounding/ Comparability (2)	Outcome	Follow-up	Adequate Follow-up	Score
Agazzi 2021[49]	*	*	*	*	*	*	*	-	7
Chang 2024[50]	*	*	*	*	**	*	*	*	9
Hur 2021[51]	-	*	*	*	**	*	*	*	8
Jimba 2021[52]	*	*	*	*	*	*	*	*	8
Jin 2022[53]	*	*	*	*	**	*	*	-	8
Jin 2023[54]	*	*	*	*	*	*	*	*	8
Kim 2021[55]	-	*	*	*	*	*	*	*	7
Kim 2023[56]	*	*	*	*	*	*	*	*	8
Liu 2019[57]	-	*	*	*	**	*	*	*	8
Nguyen 2018[58]	-	*	*	*	**	*	*	*	8
O'Sullivan 2024[59]	*	*	*	*	**	*	*	*	9
Pan 2023[60]	*	*	*	*	*	*	*	-	7
Park 2023[61]	*	*	*	*	**	*	*	*	9
Song 2023[62]	*	*	*	*	*	*	*	*	8
Syed 2019[63]	*	*	*	*	*	*	*	-	7
Wang 2022[64]	*	*	*	*	*	*	*	-	7
Yue 2021[65]	-	*	*	*	**	*	*	*	8

Cross-Sectional Studies							
	Representative	Sample Size	Exposure (2)	Adjustment for Confounders (2)	Assessment of Confounders	Outcome (2)	Score
Elangovan 2021[66]	*	*	*	**	*	*	7
Zhang 2024[67]	*	*	-	**	*	*	6

TABLE S2. PRIMARY STUDY FINDINGS.

First Author (Year) (Reference)	Location	Study Design (Period)	Age of EOCRC cohort (mean/median at diagnosis)	Sample Size (Cases)	Exposure	Exposure definition	Comparator	Assessment Method	Effect Estimate (95% CI)	Level of Adjustment
Cao (2023) [34]	Sweden	Retrospective Case-Control (1991-2017)	18 – 49 (mean 32.9)	564	Birth by caesarean section vs. by vaginal delivery	- Birth by caesarean section	- Vaginal delivery	Clinician recorded data in Electronic Health Records.	- aOR 1.28 (0.91-1.79)<br>o F: aOR 1.62 (1.01-2.60)<br>o M: aOR 1.05 (0.64-1.72)	Multivariable (matching factors, maternal and pregnancy factors)
Chang (2021) [35]	Canada	Retrospective Case-Control (2018-2019)	20 – 49 (mean 43.1)	175	Family History	- FHx CRC 1st/2nd degree relative vs not<br>- FHx CRC 1st/2nd degree relative diagnosed <50yo vs not	- No FHx	Web-based participant reported questionnaire.	- aOR 2.37 (1.47-3.84)<br>- aOR 3.35 (1.25-8.98)	Multivariate (demographic, lifestyle, dietary CRC risk factors)
					Personal History T2DM	- T2DM	- No T2DM		- aOR 1.75 (0.57-5.32)	Multivariable models as above.
					Aspirin/NSAID use	- Ever use	- Never use		- aOR 1.20 (0.75-1.92)	Multivariable models as above.
					Oral antibio-tic use	- Ever vs never<br>- Use during childhood alone (<20yo) vs never	- Never use		- aOR 0.78 (0.47-1.30)<br>- aOR 0.29 (0.09-0.90)	Multivariable models as above.
					OCP use	- Ever use	- Never use		- aOR 0.65 (0.32-1.33)	Multivariable models as above.
					Parity	- >=3 vs nulliparous<br>- First pregnancy >=30yo	- Nulliparous		- aOR 0.29 (0.11-0.76)<br>- aOR 1.90 (0.95–3.79)	Multivariable models as above.

							- First pregnancy <30yo			
					Smoking	- Ever use<br>- PYHx >=10	- Never use<br>- PYHx <10		- aOR 1.07 (0.68-1.67)<br>- aOR 0.79 (0.40-1.57)	Multivariable models as above.
					Alcohol consumption	- Daily	- <1 sd/month		- aOR 1.07 (0.49-2.32)	Multivariable models as above.
					Sedentary behaviours	- >10h/day	- <5h/day		- aOR 1.93 (1.02-3.65)	Multivariable models as above.
					Wight-based metrics	- BMI 25-29.9 kg/m2<br>- BMI >=30 kg/m2	- Normal BMI (<25 kg/m2)		- aOR 0.57 (0.34-0.94)<br>- aOR 0.59 (0.34-1.01)	Multivariable models as above.
					Vegetable and Fruit intake	- >=6 servings/day	- <3 servings/day		- aOR 0.58 (0.30-1.13)	Multivariable models as above.
					High fibre foods intake	- >=3 servings/day	- <1 servings/day		- aOR 1.45 (0.75-2.80)	Multivariable models as above.
					Red meat intake	- >=5 servings/week	- <2 servings/week		- aOR 1.06 (0.56-1.98)	Multivariable models as above.
					Processed meat intake	- >=3 servings/week	- <1 serving/week		- aOR 1.23 (0.62-2.42)	Multivariable models as above.
					Sugar sweetened beverages and desserts	- 1-6 drinks/week<br>- >=7 drinks/week<br>- 3-6 desserts/week<br>- >=7 desserts/week	- <1 drink/week<br>- <1 drink/week<br>- <3 desserts/week<br>- <3 desserts/week		- aOR 1.86 (1.11-3.13)<br>- aOR 2.99 (1.57-5.68)<br>- aOR 2.28 (1.28-4.04)<br>- aOR 1.45 (0.86-2.47)	Multivariable models as above.
Collatuzzo (2024) [36]	Iran	Retrospective Case-Control (2017-2020)	18 – 49 (mean not reported)	189	Vegetable intake	- 422-576g/day vs <422<br>- 422-576g/day in <35yo<br>- >576g/day<br>- >576g/day in <35yo	- <422g/day<br>- <422g/day<br>- <422g/day<br>- <422g/day	Validated questionnaire-based interview.	- aOR 0.58 (0.38-0.91)<br>- aOR 0.64 (0.19-2.14)<br>- aOR 0.78 (0.52-1.19)<br>- aOR 0.40 (0.10-1.58)	Multivariable-adjusted (age, province, vegetable intake, red meat

										intake, vitamin D use, family history, smoking, opium use)<br><br>For <35yo subgroup – Multivariable-adjusted (penalized regression; same covariates)
					Fruit intake	- 273.14g-429g/day<br>- 273.14g-429g/day in <35yo<br>- >429g vs <273.14g/day<br>- >429g in <35yo	- <273.14g/day<br>- <273.14g/day<br>- <273.14g<br>- <273.14g		- Crude OR 1.16 (0.78-1.73)<br>- aOR 3.36 (1.02-11.1)<br>- Crude OR 1.43 (0.98-2.09)<br>- aOR 3.67 (1.02-13.2)	Crude (unadjusted)<br><br>Multivariable models as above.
					Red meat intake	- 12.83g-25.64g/day<br>- 12.83g-25.64g/day in <35yo<br>- >35.6g/day<br>- >35.6g/day in <35yo	- <12.83 g/day<br>- <12.83 g/day<br>- <12.83 g/day<br>- <12.83 g/day		- aOR 1.04 (0.69-1.58)<br>- aOR 2.12 (0.66-6.84)<br>- aOR 1.84 (1.19-2.86)<br>- aOR 2.06 (0.46-9.14)	Multivariable models as above.
					Smoking	- Smokers	- Non-smokers		- aOR 0.97 (0.57-1.65)	Multivariable models as above.
					Family History	- FHx CRC present<br>- FHx CRC present diagnosed in <35yo	- FHx Not present<br>- FHx Not present		- aOR 1.68 (1.04-2.86)<br>- aOR 3.16 (1.29-10.9)	Multivariable models as above.
Danial (2022) [37]	USA	Retrospective Case-Control (1999-2019)	20 – 50 (mean not reported)	13800	Male Sex	- Male	- Female	Electronic Health Records database.	- aOR 1.36 (1.32-1.39)	Multivariable adjusted (demographic, metabolic, IBD, family history).

					Alcohol abuse	- Present (SNOMED-coded)	- Not present		- aOR 1.90 (1.78-2.02)	Multivariable models as above.
					Smoking	- Ever use	- Never use		- aOR 1.59 (1.54-1.65)	Multivariable models as above.
					BMI >=30 kg/m2, DM, hyperlipidaemia	- Presence of any of the three	- None of the three present		- aOR 2.49 (2.40-257)	Multivariable models as above.
					IBD	- Ulcerative Colitis present<br>- Chron's Disease present	- Not present<br>- Not present		- aOR 4.43 (4.05-4.84)<br>- aOR 3.74 (3.44-4.07)	Multivariable models as above.
					Family History	- FHx CRC present	- FHx Not present		- aOR 17.78 (16.97-18.63)	Multivariable models as above.
Gausman (2020) [38]	USA	Retrospective Case-Control (2011-2017)	18 – 49 (mean 43.0)	269	BMI	- Continuous kg/m2 (comparison of EOCRC vs Controls)	- Per unit increase	Clinician recorded data in Electronic Health Records	- 27 kg/m2 +/-6 in EOCRC vs 28 kg/m2 +/-6 in controls, p=0.06<br>- aOR 0.98 (0.95-1.00)	Multivariable adjusted (sex, family history, hyperlipidaemia)
					Family History	- FHx CRC present (comparison of EOCRC vs Controls)	- FHx not present		- 13% in EOCRC vs 2% in controls, p<0.01<br>- aOR 8.61 (4.83-15.75)	Multivariable adjusted (sex, BMI, hyperlipidaemia)
					Male sex	- Comparison of EOCRC vs Controls	- Female		- 54% EOCRC vs 45% in controls, p<0.01<br>- aOR 1.87 (1.39-2.51)	Multivariable adjusted (BMI, FHx, hyperlipidaemia)
					Smoking	- Smoker (comparison of EOCRC vs Controls)	- Non-smoker		- 27% EOCRC vs 29% in controls, p=0.53<br>- No risk ratio reported	Univariable only
					Hypertension	- Comparison of EOCRC vs Controls	- Not present		- 19% in EOCRC vs 20% in controls, p=0.85	Univariable only

					Hyper lipidae mia	- Compariso n of EOCRC vs Controls	- Not present		- 16% in EOCRC vs 23% in controls, p<0.01<br>- aOR 0.57 (0.38-0.83)	Multivaria ble adjusted (sex, BMI, FHx)
					Diabet es	- Compariso n of EOCRC vs Controls	- Not present		- 7% in EOCRC vs 6% in controls, p=0.48<br>- No risk ratio reported	Univariabl e only
Gausman (2021) [39]	UK	Prospec tive Case-Control (2006-2019)	38 – 49 (mea n not report ed)	455 pre val ent cas es 85 inci den t cas es	Breastf ed	- Breastfed	- Not breastfed	Electro nic Health Record . UK Bioban k.	- aOR 1.04 (0.84-1.29)	Multivaria ble adjusted (age, race, sex, household income, family history of CRC, other early factors)*No t adjusted for sex where sex-specific
					Comp arative body size to peers at 10 years	- Average<br>- Plumper	- Thinn er<br>- Thinn er		- aOR 1.04 (0.85-1.26)<br>- aOR 1.13 (0.87-1.45)	Multivaria ble model as above.
					Comp arative height to peers at 10 years	- Shorter<br>- Taller	- Avera ge<br>- Avera ge		- aOR 1.21 (0.96-1.53)<br>- aOR 1.13 (0.87-1.48)	Multivaria ble model as above.
					Mater nal smoki ng at birth	- Maternal smoking at birth present	- Not present		- aOR 0.94 (0.77-1.15)	Multivaria ble model as above.
					Age of menar che in F	- 12-13yo<br>- >=14yo	- <11yo<br>- <11yo		- aOR 1.13 (0.82-1.59)<br>- aOR 1.19 (0.85-1.68)	Multivaria ble model as above.
					Relati ve age of first	- Younger<br>- Older	- Avera ge		- aOR 0.80 (0.54-1.23)	Multivaria ble model as above.

					facial hair in M			-	Average		-	aOR 0.64 (0.38-1.10)	
Glover (2019) [40]	USA	Retrospective Case-Control (2013-2018)	20 – 39 (mean not reported)	1680	Male Sex	-	Male	-	Female	Clinician recorded data in Electronic Health Records. SNOMED-CT	-<br>-	Crude OR 1.29 (1.17-1.41)<br>aOR 0.27 (0.25-0.30)	Crude (unadjusted) Multivariable adjusted (race, DM, smoking, obesity, alcohol, FHx non-CRC)
					Race	-	Caucasian	-	Non-Caucasian		-	aOR 1.73 (1.55-1.92)	Multivariable model as above.
					Diabetes	-	Present (SNOMED-coded)	-	Not present		-	aOR 19.80 (18.15-21.60)	Multivariable model as above.
					Smoking	-	Smoker	-	Non-smoker		-	aOR 2.68 (2.41-2.97)	Multivariable model as above.
					Alcohol	-	Alcohol abuse present (SNOMED-coded)	-	Not present		-	aOR 0.91 (0.66-1.25)	Multivariable model as above.
					Family History	-	FHx of other cancer NOT CRC present	-	Not present		-	aOR 7.33 (6.18-8.70)	Multivariable model as above.
Low (2020) [41]	USA	Retrospective Case-control (1999-2014)	18 – 49 (mean 44.8)	651	Age	-	Per 1-year increase at colonoscopy	-	Younger age	Clinician recorded data in Electronic Health Records.	-	aOR 1.05 (1.03-1.07)	Multivariable adjusted (sex, smoking, aspirin, BMI)
					Male sex	-	Male	-	Female		-	aOR 2.21 (1.68-2.91)	Multivariable model as above.
					Smoking	-<br>-	Former smoker<br>Current smoker	-<br>-	Never smoker<br>Never smoker		-<br>-	aOR 0.82 (0.60-1.12)<br>aOR 1.10 (0.89-1.35)	Multivariable model as above.
					Aspirin use	-	Ever use	-	Never use		-	aOR 0.66 (0.52-0.84)	Multivariable model as above.

					Weight-based metric (no BMI references)	- Underweight<br>- Overweight<br>- Obese	- Normal<br>- Normal<br>- Normal		- aOR 1.87 (0.86-4.04)<br>- aOR 0.69 (0.55-0.87)<br>- aOR 0.69 (0.55-0.86)	Multivariable model as above.
Martel-Martel (2023) [42]	Spain	Prospective Case-control (2022)	18 – 49 (mean not reported)	87 (70 for genetic analysis)	Leukocyte telomere length	- Absolute Telomere length (kb) mean in EOCRC	Absolute Telomere length (kb) mean in controls	Clinician evaluated. Quantified using Real-Time Quantitative PCR (RT-qPCR)	- EOCRC TL mean 122kb vs controls TL mean 296kb, p<0.001<br>- No risk ratios reported	Crude comparison (t-test only)
Nguyen (2022) [43]	Sweden	Retrospective Case-control (2006-2016)	18 – 49 (mean 42.9)	2557	Antibiotic use	- >=1 ABx use before 6-month lag<br>- >=1 broad-spectrum ABx use<br>- >=1 narrow-spectrum ABx use<br>- >=1 Anti-aerobic ABx us<br>- >=1 Anti-anaerobic ABx use	- No ABx use<br>- No broad-spectrum ABx use<br>- No narrow-spectrum ABx use<br>- No anti-aerobic ABx use<br>- No anti-anaerobic ABx use	Clinical record data in Pharmacy Registry.	- aOR 1.06 (0.96-1.17)<br>- aOR 1.13 (1.02-1.26)<br>- aOR 1.01 (0.92-1.11)<br>- aOR 1.07 (0.96-1.17)<br>- aOR 1.01 (0.87-1.17)	Multivariable Conditional logistic regression (education, healthcare utilisation, CCI, prior endoscopy)
Puzzono (2022) [44]	Italy	Retrospective Case-control (2018-2020)	18 – 49 (mean not reported)	47	Family History	- FHx CRC in first degree relative (FDR) in CRC cohort	- Controls	Participant-reported questionnaire.	- 7 in EOCRC vs 1 in controls, p=0.004<br>-	Crude comparison (X^2 test only)
					Smoking	- Never in CRC cohort<br>- <10cig/day in CRC cohort<br>- >10cig/day in CRC cohort	- Controls		- 57.4% in EOCRC vs 78.9% in controls, p=0.0001<br>- 7.6% in EOCRC vs 8.5% in controls, p=0.0001	Crude comparison (X^2 test only)

									- 6.4% in EOCRC vs 11.2% in controls, p=0.0001<br>-	
					Physical activity	- Never in CRC cohort<br>- <1/week in CRC cohort<br>- 1/week in CRC cohort<br>- >1/week in CRC cohort	- Controls		- 42.6% in EOCRC vs 35.2% in controls, p=0.76<br>- 6.4% in EOCRC vs 7.0% in controls, p=0.76<br>- 10.6% in EOCRC vs 16.9%, in controls, p=0.76<br>- 40.4% in EOCRC vs 40.9% in controls, p=0.76	Crude comparison (X² test only)
					Fresh meat intake (for 5-years prior to dx)	- Median intake in CRC cohort	- Controls		- 4-5/week in EOCRC vs 2-3/week in controls, p=0.001<br>-	Crude median test only
					Processed meat intake (for 5-years prior to dx)	- Median intake in CRC cohort	- Controls		- Median 4-5/week in EOCRC vs 2-3/week in controls, p<0.001<br>-	Crude median test only
Chen (2021) [45]	USA	Retrospective Nested Case-Control (2006-2015)	18 – 49 (mean 43.0)	4673	Metabolic Syndrome	- Refer to Table 2 for definition and comorbid conditions (ICD-9). Present.	- Not present	Claims-based longitudinal database.	- aOR 1.25 (2.09-2.43)	Multivariable adjusted (demographics, insurance, comorbidities, screening, healthcare utilisation)
					No. of comorbid conditions.	- 1<br>- 2<br>- >=3	- 0 conditions<br>- 0 conditions<br>- 0 conditions		- aOR 1.09 (1.00-1.17)<br>- aOR 1.12 (1.01-1.24)<br>- aOR 1.31 (1.13-1.51)	Multivariable model as above.
Kane (2024) [46]	USA	Retrospective Nested Case-	18 – 49 (mean not	1359	Antibiotic use (>1 year	- Any use >=1 prescription	- No antibiotic use	Electronic Health Record	- aOR 1.04 (0.90-1.22)	Multivariable conditional logistic

	control (1998-2020)	reported)	before index date)			. Pharmacy dispensing records.		regression (age, sex, race, primary care facility, BMI, smoking)
				- Broad spectrum antibiotic use<br>- Narrow spectrum antibiotic use<br>- Anti-aerobic antibiotic use<br>- Anti-anaerobic antibiotic use	- No antibiotic use		- aOR 1.08 (0.91-1.28)<br>- aOR 1.05 (0.90-1.22)<br>- aOR 1.04 (0.90-1.22)<br>- aOR 1.03 (0.90-1.18)	Multivariable conditional logistic regression (age, sex, race, primary care facility, BMI, smoking)
				- Penicillin use<br>- Tetracycline use<br>- Macrolide use<br>- Quinolone use<br>- Sulfonomide use<br>- Cephalosporin use<br>- Other class of antibiotic use	- No antibiotic use		- aOR 0.98 (0.86-1.13)<br>- aOR 1.03 (0.87-1.23)<br>- aOR 1.13 (0.97-1.31)<br>- aOR 1.01 (0.84-1.20)<br>- aOR 0.95 (0.81-1.11)<br>- aOR 0.99 (0.85-1.15)<br>- aOR 1.15 (0.90-1.46)	Multivariable conditional logistic regression (age, sex, race, primary care facility, BMI, smoking)
				- Antibiotic no. of exposures 1-3 vs 0<br>- Antibiotic no. of exposures 4-6 vs 0<br>- Antibiotic no. of exposures 7-9 vs 0<br>- Antibiotic no. of exposures >9 vs 0<br>- Antibiotic interval 2-4 years vs never<br>- Antibiotic interval 5-7 years vs never	- 0 dispensations		- aOR 1.07 (0.92-1.25)<br>- aOR 1.06 (0.85-1.32)<br>- aOR 1.07 (0.80-1.44)<br>- aOR 1.15 (0.87-1.54)<br>- aOR 1.01 (0.78-1.30)<br>- aOR 1.07 (0.83-1.39)<br>- aOR 0.88 (0.64-1.21)	Multivariable conditional logistic regression (age, sex, race, primary care facility, BMI, smoking)

						- Antibiotic interval >=8 years vs never				
Li (2022) [47]	USA	Retrospective Nested Case-Control (2006-2015)	18 – 49 (mean 43.0)	6001	Diabetes	- T2DM present (ICD-9)<br>- T2DM (Controlled ICD-9)<br>- T2DM (uncontrolled ICD-9)<br>- T2DM (complicated ICD-9)	- T2DM not present<br>- T2DM not present<br>- T2DM not present<br>- T2DM not present	Claims based longitudinal database.	- aOR 1.24 (1.09-1.41)<br>- aOR 1.13 (0.94-1.36)<br>- aOR 1.37 (1.12-1.67)<br>- aOR 1.59 (1.08-2.35)	Multivariable adjusted (demographic, insurance, comorbidities, screening, symptoms)
Lundqvist (2023) [48]	Sweden	Retrospective Nested Case-Control (2007-2016)	18 – 49 (mean 42.5)	2626	Metabolic disease	- Metabolic disease without IBD<br>- Metabolic disease with IBD<br>- Refer to table 2 for definitions (ICD-10)	- No metabolic disease	Clinician recorded data in Electronic Health Records.	- aHR 1.28 1.08-1.52)<br>- aHR 3.65 (2.57-5.19)	Conditional logistic regression (matched on age, sex, county); limited additional covariate adjustment
Agazzi (2021) [49]	Italy	Retrospective Cohort (2015-2018)	18 – 49 (mean 42.5)	27	Sex	- Female	- Male	Electronic Health Records and endoscopy database.	- aOR 0.59 (0.27-1.29)	Multivariable adjusted (age, sex, alarm symptoms, GI symptoms, smoking, IBD, family history)
					Family History	- FHx of CRC 1st to 3rd degree relative	- No family history		- aOR 0.76 (0.22-2.58)	Age-adjusted logistic regression only (due to small sample size)
Chang (2024) [50]	South Korea	Retrospective Cohort (2009-2011)	20 – 49 (mean not reported)	7492	Hypertriglyceridaemia (fasting)(ICD-10)	- Quartile 1 < 71 mg/dL = reference<br>- Q2: 71-104 mg/dL vs reference<br>- Q3: 105-160 mg/dL vs reference<br>- Q4: ≥ 161 mg/dL vs reference	- Reference<br>- <71mg/dL<br>- <71mg/dL<br>- <71mg/dL<br>- Reference	Serum measured TG level recorded in Health Database.2 measur	- 1.0 (reference group)<br>- aHR 1.07 (1.00-1.14)<br>- aHR 1.12 (1.04-1.20)<br>- aHR 1.15 (1.06-1.23)<br>- 1.0 (reference group)	Multivariable adjusted (demographics, lifestyle, metabolic factors)

						- Persistent Normal-TG = reference<br>- Normal-TG change to Hyper-TG<br>- Hyper-TG to Normal-TG.<br>- Persistent Hyper-TG	- Persistent normal TG<br>- Persistent normal TG<br>- Persistent normal TG	ements (2009, 2011)	- aHR 1.01 (0.93-1.09)<br>- aHR 1.01 (0.93-1.10)<br>- aHR 1.10 (1.03-1.17)	
Hur (2021) [51]	USA	Prospective Cohort (1991-2015 recruitment)	25 – 42 at enrolment (mean not reported)	109	Sugar Sweetened Beverages	- <1 serving/day – 1 serving/week<br>- 1-<2 servings/day<br>- >=2 servings /day<br>- Per 1 serving/day increase	- <1 serving (12 oz)/week<br>- <1 serving (12 oz)/week<br>- <1 serving (12 oz)/week	Participant reported questionnaires. NHSII.	- aRR 0.97 (0.61-1.55)<br>- aRR 1.24 (0.65-2.39)<br>- aRR 2.18 (1.10-4.35)<br>- aRR 1.16 (1.00-1.36)	Multivariable adjusted (Cox proportional hazards; demographics, BMI, lifestyle, diet quality, total energy intake, and screening/endoscopy history).
Jimba (2021) [52]	Japan	Retrospective Cohort (2005-2018)	20 – 49 (mean not reported)	1884	Metabolic Syndrome	- Refer to Table 2 for definition and comorbid conditions. Present vs not.<br>o Male<br>o Female	- No metabolic syndrome	Clinician recorded data in Electronic Health Records.	- aHR 1.26 (1.07-1.49)<br>o M: aHR 1.26 (1.05-1.50)<br>o F: aHR 1.27 (0.77-2.10)	Multivariable adjusted (Cox proportional hazards; demographics + metabolic/lifestyle covariates)
Jin (2022) [53]	South Korea	Retrospective Cohort (2009-2010, follow up to 2019)	20 – 49 (median 46)	8320	Waist circumference	- Male: >=90 cm and Female: >=85cm	- Male: <90cm and<br>- Female: <85cm	Clinician recorded measurement.	- aHR 1.23 (1.16-1.30)	Multivariable Cox proportional hazards regression model (adjusted for demographics and lifestyle factors)

					BMI (kg/m2)	- <18.5<br>- 23-24.9<br>- 25-29.9<br>- >=30	- 18.5-22 kg/m2		- aHR 1.00 (0.88-1.14)<br>- aHR 1.10 (1.04-1.17)<br>- aHR 1.19 (1.12-1.25)<br>- aHR 1.45 (1.31-1.61)	Multivariable model as above.
					Fasting glucose	- High	- Normal		- aHR 1.08 (1.03-1.13)	Multivariable model as above.
					Blood pressure	- High (>=140/90 mmHg)	- Normal		- aHR 1.13 (1.07-1.18)	Multivariable model as above.
					Triglycerides	- High (>=150mg/dL)	- <150mg/dL		- aHR 1.13 (1.08-1.18)	Multivariable model as above.
					Metabolic Syndrome Comorbidities	- >=3 components present<br>- 1<br>- 2<br>- 3<br>- 4<br>- 5<br>- Refer to Table 2 for definitions	- 0 metabolic components		- aHR 1.20 (1.14-1.27)<br>- aHR 1.07 (1.01-1.13)<br>- aHR 1.13 (1.06-1.21)<br>- aHR 1.25 (1.16-1.35)<br>- aHR 1.27 (1.15-1.41)<br>- aHR 1.50 (1.26-1.79)	Multivariable model as above.
Jin (2023) [54]	South Korea	Retrospective Cohort (2009-2019)	20 – 49 (mean not reported)	8314	Alcohol intake.	- Non-drinker (0g/day)<br>o Male<br>o Female<br>- Mod-drinker (10-<30g/day) vs light<br>o Male<br>o Female<br>- Heavy-drinker (>=30g/day) vs light<br>o Men<br>o Women	- Light drinkers (<10g/day)	Participant reported questionnaire. NHIS.	- aHR 0.96 (0.91-1.02)<br>o M: aHR 0.99 (0.91-1.06)<br>o F: aHR 0.94 (0.86-1.02)<br>- aHR 1.09 (1.02-1.16)<br>o M: aHR 1.09 (1.02-1.17)<br>o F: aHR 1.11 (0.94-1.32)<br>- aHR 1.20 (1.11-1.29)<br>o M: aHR 1.21 (1.11-1.31)<br>o F: aHR 1.20 (0.97-1.47)	Multivariable adjusted (age, sex, smoking, regular exercise, low income, hypertension, diabetes, dyslipidaemia)
Kim (2021) [55]	USA	Prospective Cohort (1991-	25 – 42 at enrolment	111	Vitamin D intake	- Total Vit D intake per day<br>o 300-<450<br>o >=450	- <300IU/day	Participant reported	- Reference (<300IU/day)<br>o 300-< 450: aHR 0.51 (0.30-0.86)	Multivariable adjusted (demograp

		2015 recruitment)	(mean not reported)			- Total Vit D intake, per 400IU/day increase<br>- Total Vit D dietary intake vs <150IU/day<br>o 150-<300<br>o >=300<br>- Total Vit D dietary intake, per 400IU/day increase<br>- Total Vit D supplement intake vs <150IU/day<br>o 150-<300<br>o >=300<br>- Total Vit D supplement intake, per 400IU/day increase		questionnaires. NHSII.	o >=450: aHR 0.49 (0.26-0.93)<br>- Per 400IU/day increase: aHR 0.46 (0.26-0.83)<br>- Reference (<150IU/day)<br>o 150-<300: aHR 0.75 (0.48-1.17)<br>o >=300: aHR 0.50 (0.27-0.93)<br>- Per 400IU/day increase: aHR 0.34 (0.15-0.79)<br>- Reference (150IU/day)<br>o 150-<300: aHR 0.90 (0.51-1.60)<br>o >=300: aHR 0.64 (0.28-1.45)<br>- aHR 0.77 (0.37-1.62)	hics, BMI, smoking, alcohol, physical activity, diet quality, screening history)
Kim (2023) [56]	South Korea	Retrospective Cohort (2011-2018)	18 – 49 (median 42.4 amongst incident cases)	236 382	Vitamin D level	- 10-19 ng/mL<br>- >=20 ng/mL	<10ng/mL	Serum measured 25(OH) D level (ng/ml)	- aHR 0.61 (0.43-0.86)<br>- aHR 0.41 (0.27-0.63)	Multivariable time-dependent Cox model (demographic, lifestyle, metabolic and supplement factors)
Liu (2019) [57]	USA	Prospective Cohort (1989-2011 recruitment)	25 – 42 at enrolment (median 45)	114	Weight-based metrics	- Current BMI (kg/m2)<br>o 23-24.9<br>o 25-29.9<br>o >=30<br>- Each 5 unit increase in BMI<br>- BMI (kg/m2) at 18yo<br>o <18.5<br>o 21.0-22.9<br>o >=23<br>o BMI each 5-unit increase<br>- Weight change since 18yo<br>o Gain 5-19kg	- 18.5-22.9 kg/m2<br>- 18.5-22.9 kg/m2<br>- Loss or gain of <5kg	Participant reported questionnaires. NHSII.	- Reference (18.5-22.9 kg/m2)<br>o 23-24.9: aRR 1.33 (0.75-2.36)<br>o 25-29.9: aRR 1.37 (0.81-2.30)<br>o >=30: aRR 1.93 (1.15-3.25)<br>- Per 5-unit increase: aRR 1.20 (1.05-1.38)<br>- Reference (18.5-22.9 kg/m2)<br>o <18,5: aRR 1.05 (0.56-1.97)<br>o 21.0-22.9: aRR 1.32 (0.80-2.16)<br>o >=23: aRR 1.63 (0.81-1.40)	Multivariable adjusted (height, family history, diabetes, screening history, endoscopy, smoking, alcohol, medication use, physical activity, diet)

						○ Gain 20-39.9kg<br>○ Gain >=40kg<br>○ Each 5kg increase			○ Per 5-unit increase: aRR 1.06 (0.81-1.40)<br>- Reference (loss/gain <5kg)<br>○ Gain 5-19kg: aRR 0.86 (0.52-1.43)<br>○ Gain 20-39.9kg: aRR 1.65 (0.96-2.81)<br>○ Gain >=40kg: aRR 2.15 (1.01-4.55)<br>○ Per 5kg increase: aRR 1.09 (1.02-1.16)	
Nguyen (2019) [58]	USA	Prospective Cohort (1991-2011 recruitment)	25 – 42 (median 45)	118	Sedentary behaviours (TV viewing time)	- 7.1-14h/week<br>- >14h/week	- <=7h/week	Participant reported questionnaires. NHSII.	- aRR 1.12 (0.72-1.75)<br>- aRR 1.69 (1.07-2.67)	Multivariable adjusted (demographics, BMI, lifestyle factors, screening history, and dietary variables)
O'Sullivan (2024) [59]	Canada	Prospective Cohort (OHS 2009-2017, ATP 2000-2015)	18 – 49 (mean not reported)	98	Sex	- Female	- Male	Participant-reported questionnaire.	- aHR 1.02 (0.64-1.64)	Multivariable adjusted (age as time scale) (demographics, socioeconomic status, BMI/WC, smoking, alcohol, physical activity, comorbidities, diet, and family history; pooled using random-effects meta-analysis)

					Joint BMI and Waist-circumference	- high BMI (>=25 kg/m2) and normal waist circumference<br>- High BMI and waist circumference (>=80cm in F and >=94 in M)	- Normal BMI and waist circumference		- aHR 1.03 (0.44-2.42)<br>- aHR 0.96 (0.58-1.57)	Multivariable model as above.
					Smoking	- current heavy >=10PYHx<br>- current light <10PYHx<br>- Former heavy<br>- Former light	- Never smoker		- aHR 1.87 (1.00-3.52)<br>- aHR 1.13 (0.25-5.16)<br>- aHR 1.21 (0.56-2.63)<br>- aHR 1.23 (0.72-2.11)	Multivariable model as above.
					Alcohol intake	- Monthly drinker<br>- Weekly drinker<br>- Daily drinker	- Non-drinker		- aHR 1.59 (0.65-3.87)<br>- aHR 1.72 (0.73-4.04)<br>- aHR 1.72 (0.73-4.04)	Multivariable model as above.
					Diabetes	- Present	- Not present		- aHR 0.90 (0.28-2.93)	Multivariable model as above.
					High Blood Pressure	- Present	- Not present		- aHR 1.10 (0.59-2.08)	Multivariable model as above.
					Physical activity	- MET-hr/week vs low<br>○ Medium<br>○ High	- Low MET-hr/week tertile		- Reference (Low MET-hr/week)<br>○ Medium: aHR 1.15 (0.54-2.45)<br>○ High: aHR 1.18 (0.70-1.8)	Multivariable model as above.
					Family History	- FHx of CRC<br>- FHx non-CRC only	- No FHx		- aHR 2.76 (1.43-5.32)<br>- aHR 1.18 (0.61-2.30)	Multivariable model as above.
					Fruit and Vegetable intake	- 8 servings/day	- <8 servings/day		- aHR 0.79 (0.43-1.43)	Multivariable model as above.
					Parity	- Yes	- Nulliparous		- aHR 0.76 (0.34-1.73)	Multivariable model as above.
Pan (2023) [60]	China	Prospective Cohort (2004-	30 – 50 (medi	222	Weight-based metric	- BMI >=28 kg/m2<br>○ Male<br>○ Female	- Normal (18.5-23.9 kg/m2)	Questionnaire based	- aHR 1.04 (1.00-1.08)<br>○ M: aHR 1.02 (0.95-1.09)	Multivariable adjusted (age, sex,

	2008 recruitment)	an 46.3)				interview	o F: aHR 1.07 (1.01-1.12)	region, BMI, smoking, alcohol, diet, hypertension, diabetes, and family history of cancer).
			Smoking	- Ex-regular smoker<br>- Regular smoker	- Never smoker		- aHR 0.97 (0.46-2.06)<br>- aHR 1.69 (1.12-2.91)	Multivariable model as above.
			Alcohol intake	- Ex-regular drinker<br>- Regular drinker	- Never drinker		- aHR 3.54 (1.10-11.35)<br>- aHR 1.69 (1.12-2.91)	Multivariable model as above.
			Fish intake	- Monthly<br>- Weekly	- Never		- aHR 1.81 (1.12-2.91)<br>- aHR 1.64 (1.01-2.67)	Multivariable model as above.
			Red meat intake	- 1-6d/week<br>- Daily	- Never		- aHR 1.24 (0.78-1.97)<br>- aHR 0.91 (0.53-1.57)	Multivariable model as above.
			Poultry intake	- 1-6d/week<br>- Daily	- Never		- aHR 1.06 (0.70-1.61)<br>- aHR 1.04 (0.66-1.65)	Multivariable model as above.
			Egg intake	- 4-6d/week<br>- 1-3d/week<br>- Monthly<br>- Never	- Daily		- aHR 1.09 (0.66-1.81)<br>- aHR 0.92 (0.61-1.40<br>- aHR 1.13 (0.71-1.80)<br>- aHR 0.57 (0.28-1.16)	Multivariable model as above.
			Hypertension	- Present<br>o Male<br>o Female	- Not present		- aHR 1.99 (1.04-3.81)<br>o M: aHR 1.69 (0.60-4.674)<br>o F: aHR 2.32 (1.01-5.34)	Multivariable model as above.
			DM	- Present<br>o Male<br>o Female	- Not present		- aHR 2.20 (1.08-4.49)<br>o M: aHR 1.80 (0.56-5.76)<br>o F: aHR 2.62 (1.06-6.48)	Multivariable model as above.

					Family history	- FHx in first-degree relative (FDR) of any cancer<br>○ Male<br>○ Female	- Not FDR with cancer		- aHR 1.70 (1.23-2.36)<br>○ M: aHR 2.32 (1.43-3.75)<br>○ F: aHR 1.32 (0.83-2.08)	Multivariable model as above.
Park (2023) [61]	South Korea	Retrospective Cohort (2009-2012)	20 – 39 (mean not reported)	7910	Metabolic conditions	- CRC in NAFLD (based on international guidelines of European association for study of liver)	- No NAFLD	National health screening.	- aHR 1.14 (1.06-1.22)	Multivariable adjusted (age, sex, BMI, smoking, alcohol use, physical activity, income, diabetes, pancreatitis, and cholangitis)
Song (2023) [62]	South Korea	Retrospective Cohort (2009-2011)	20 – 49 (mean not reported)	7492	Weight-related metrics.	- No obesity on 1st, but yes on 2nd exam<br>- Obesity on 1st, but not on 2nd exam<br>- Obesity on both exams<br>- Obesity and Abdominal Adiposity (>90cm in M, >85cm in F)<br>- Normal AA in first, but high in 2nd exam<br>- High AA in first, but not 2nd exam<br>- High AA in both exams	- No obesity on either exam<br>- Not present<br>- Normal AA both exams	Clinician measured. NHIS. 2 examinations 2009 and 2011.	- aHR 0.97 (0.88-1.08)<br>- aHR 1.08 (0.96-1.21)<br>- aHR 1.09 (1.03-1.30)<br>- aHR 1.19 (1.09-1.30)<br>- aHR 1.08 (0.98-1.19)<br>- aHR 1.11 (1.00-1.23)<br>- aHR 1.18 (1.09-1.29)	Multivariable adjusted (age, sex, smoking, alcohol use, exercise, income, fasting glucose, and total cholesterol)
Syed (2019) [63]	USA	Retrospective Cohort (2012-2016)	24 – 49 (mean not reported)	5710	Sex	- Male<br>- Female	- Opposite sex	Clinician recorded Electronic Health Record.	- aOR 1.34 (1.27-1.41)<br>- aOR 0.75 (0.71-0.79)	Multivariable adjusted (demographics, family history variables, lifestyle factors, comorbidities, personal

										polyp history, and CRC-related symptoms)
					Race	- Caucasian<br>- African American<br>- Asian	- Individuals without CRC in same group without exposure.		- aOR 1.48 (1.40-1.57)<br>- aOR 1.25 (1.17-1.35)<br>- aOR 0.88 (0.72-1.06)	Multivariable model as above.
					Family History	- FHx any cancer<br>- FHx gastrointestinal malignancy<br>- FHx polyps	- No FHx		- aOR 11.66 (10.97-12.39)<br>- aOR 28.67 (26.64-30.86)<br>- aOR 8.15 (6.31-10.52)	Multivariable model as above.
					Smoking	- Tobacco use	- No tobacco use		- aOR 2.36 (2.33-2.59)	Multivariable model as above.
					Alcohol intake	- Ever alcohol use	- Never use		- aOR 1.71 (1.62-1.8)	Multivariable model as above.
					Weight-based metric	- BMI >=30 kg/m2	- BMI <30 kg/m2		- aOR 2.88 (2.74-3.04)	Multivariable model as above.
					Hypertension	- Present	- Not present		- aOR 2.86 (2.70-3.03)	Multivariable model as above.
					Hyperlipidaemia	- Present	- Not present		- aOR 2.39 (2.23-2.55)	Multivariable model as above.
Wang (2022) [64]	USA	Retrospective Cohort (2010-2021)	20 – 49 (mean not reported)	571 (with diverticular disease), 300 (without diverticular	Diverticular Disease.	- Present (ICD-10)	- Not present	Clinician recorded Electronic Health Record.	- aOR 1.76 (1.40-2.32)	Propensity-score matched on age, sex, race/ethnicity, socioeconomic factors, lifestyle, obesity, NSAIDs, aspirin, prior digestive malignancy (same model across

				disease)						study years)
Yue (2021) [65]	USA	Prospective Cohort (1981-2015)	25 – 42 (mean 45)	111	Diet	- Prime diet quality score<br>- Overall plant-based diet index<br>- Empirical dietary index for hyperinsulinaemia<br>- Empirical lifestyle index for hyperinsulinaemia (ELIH)	- Quartile 4 vs Quartile 1<br>- Quartile 4 vs Quartile 1<br>- Quartile 4 vs Quartile 1<br>- Quartile 4 vs Quartile 1	Participant reported questionnaires. NHSII.	- aHR 0.90 (0.55-1.50)<br>- aHR 1.24 (0.74-2.08)<br>- aHR 1.24 (0.72-2.16)<br>- aHR 1.86 (1.12-3.07)	Multivariable adjusted (age, alcohol, height, race, BMI, family history, diabetes, smoking, physical activity, medication, history of endoscopy) *ELIH - adjustment excluded BMI and physical activity
Elangovan (2021) [66]	USA	Cross-Sectional (2017-2021)	20 – 50 (mean not reported)	16090	Weight-related metric	- BMI >=30 kg/m2<br>o 20-39yo M<br>o 20-39yo F<br>o 40-49yo M<br>o 40-49yo F	- BMI <30 kg/m2	Clinical recorded data in Electronic Health Record. SNOMED-CT definitions.	- aOR 1.92 (1.85-1.99)<br>- aOR 2.22 (1.84-2.43)<br>- aOR 1.96 (1.87-2.06)<br>- aOR 1.49 (1.41-1.57)	Multivariable adjusted (sex, race, obesity, diabetes, hypertension, hyperlipidaemia, smoking)
					Diabetes	- T2DM<br>o 20-39yo M<br>o 20-39yo F<br>o 40-49yo M<br>o 40-49yo F	- No T2DM		- aOR 3.42 (2.85-5.37)<br>- aOR 0.95 (0.84-1.07)<br>- aOR 2.00 (1.75-2.28)<br>- aOR 0.76 (0.68-1.05)	Multivariable model as above.
					Hypertension	- Hypertension<br>o 20-39yo M<br>o 20-39yo F<br>o 40-49yo M	- No hypertension		- aOR 3.43 (2.77-4.22)<br>- aOR 1.00 (0.93-1.07)	Multivariable model as above.

						o 40-49yo F			- aOR 2.00 (1.85-2.15)<br>- aOR 1.27 (1.13-1.42)	
					Hyperlipidaemia	- Hyperlipidaemia<br>o 20-39yo M<br>o 20-39yo F<br>o 40-49yo M<br>o 40-49yo F	- No hyperlipidaemia		- aOR 3.42 (2.48-4.04)<br>- aOR 2.37 (2.22-2.52)<br>- aOR 1.50 (1.35-1.67)<br>- aOR 1.47 (1.32-1.63)	Multivariable model as above.
					Smoking	- Smoker ever<br>o 20-39yo M<br>o 20-39yo F<br>o 40-49yo M<br>o 40-49yo F	- Never smoker		- aOR 1.77 (1.43-2.15)<br>- aOR 0.96 (0.91-1.00)<br>- aOR 1.25 (1.16-1.36)<br>- aOR 1.16 (1.08-1.25)	Multivariable model as above.
Zhang (2024) [67]	USA	Cross-sectional (2004-2018)	18 – 49 (mean 41.8)	156	Age	- Increasing age	- Per 1-year increase	Participant reported data. NHIS.	- aOR 1.11 (1.08-1.14)	Multivariable adjusted (age, sex, race/ethnicity, region, BMI, diabetes, alcohol status, smoking status, and physical activity)
					Sex	- Female	- Male		- aOR 0.83 (0.54-1.26)	Multivariable model as above.
					Race	- Hispanic<br>- Non-Hispanic black<br>- Non-Hispanic Asian	- Non-Hispanic white		- aOR 0.43 (0.22-0.84)<br>- aOR 0.77 (0.44-1.33)<br>- aOR 0.38 (0.16-0.92)	Multivariable model as above.
					Weight-based metric	- BMI <25 kg/m2<br>- BMI 25-<30 kg/m2	- BMI >=30 kg/m2		- aOR 1.01 (0.57-1.79)<br>- aOR 1.04 (0.64-1.68)	Multivariable model as above.
					Alcohol intake	- Former drinker<br>- Current irregular/light	- Never drinker		- aOR 2.09 (1.01-4.36)<br>- aOR 0.84 (0.45-1.55)	Multivariable model as above.

		- Current moderate		- aOR 1.23 (0.63-2.40)	
	Smoking	- Current smoker<br>- Former smoker	- Never smoker	- aOR 0.79 (0.47-1.33)<br>- aOR 1.01 (0.58-1.76)	Multivariable model as above.
	Physical activity	- Vigorous exercise<br>- Moderate to vigorous exercise	- Never exercised	- aOR 0.34 (0.21-0.55)<br>- aOR 0.58 (0.34-1.00)	Multivariable model as above.

PRISMA ABSTRACT CHECKLIST [30]

Section and Topic	Item #	Checklist item	Reported (Yes/No)	Details
**TITLE**				
Title	1	Identify the report as a systematic review.	Y	Risk factors in Sporadic Early-Onset Colorectal Cancer, current evidence and emerging insights: A Systematic Review.
**BACKGROUND**				
Objectives	2	Provide an explicit statement of the main objective(s) or question(s) the review addresses.	Y	To update and synthesise the current body of evidence on modifiable and non-modifiable risk factors for sporadic early-onset colorectal cancer.
**METHODS**				
Eligibility criteria	3	Specify the inclusion and exclusion criteria for the review.	Y	Eligibility Criteria included in methods. Further details included in Supplementary material.
Information sources	4	Specify the information sources (e.g. databases, registers) used to identify studies and the date when each was last searched.	Y	Details are outlined in methods. "A search strategy developed in consultation with an academic librarian at University of New South Wales (UNSW) was applied to PubMed and EMBASE databases from inception to 1 March 2025." Search strategy included in Supplementary Material.

Section and Topic	Item #	Checklist item	Reported (Yes/No)	Details
Risk of bias	5	Specify the methods used to assess risk of bias in the included studies.	Y	Details are provided in methods. Assessments are provided in supplementary materials. S1.
Synthesis of results	6	Specify the methods used to present and synthesise results.	Y	Details outlined in Data Synthesis Methods section. SWiM methodology referenced.
**RESULTS**				
Included studies	7	Give the total number of included studies and participants and summarise relevant characteristics of studies.	Y	Details are outlined in results Study Characteristics. Outlined in Table 1.
Synthesis of results	8	Present results for main outcomes, preferably indicating the number of included studies and participants for each. If meta-analysis was done, report the summary estimate and confidence/credible interval. If comparing groups, indicate the direction of the effect (i.e. which group is favoured).	Y	Details outlined in Results section, under relevant categories.
**DISCUSSION**				
Limitations of evidence	9	Provide a brief summary of the limitations of the evidence included in the review (e.g. study risk of bias, inconsistency and imprecision).	Y	Discussed in Discussion and reported in separate Limitations section.
Interpretation	10	Provide a general interpretation of the results and important implications.	Y	Provided in Conclusions.

Section and Topic	Item #	Checklist item	Reported (Yes/No)	Details
**OTHER**				
Funding	11	Specify the primary source of funding for the review.	Y	Nil funding obtained.
Registration	12	Provide the register name and registration number.	Y	[PROSPERO (CRD420251063020)].

PRISMA MANUSCRIPT CHECKLIST [30]

Section and Topic	Item #	Checklist item	Location where item is reported
**TITLE**			
Title	1	Identify the report as a systematic review.	Title - Risk factors in Sporadic Early-Onset Colorectal Cancer, current evidence and emerging insights: A Systematic Review.
**ABSTRACT**			
Abstract	2	See the PRISMA 2020 for Abstracts checklist.	Abstract Checklist attached separately.
**INTRODUCTION**			
Rationale	3	Describe the rationale for the review in the context of existing knowledge.	Outlined in 'Introduction'.
Objectives	4	Provide an explicit statement of the objective(s) or question(s) the review addresses.	Outlined in 'Introduction' and 'Methods'. Objective: To update and synthesise the current body of evidence on modifiable and non-modifiable risk factors for sporadic early-onset colorectal cancer.
**METHODS**			
Eligibility criteria	5	Specify the inclusion and exclusion criteria for the review and how studies were grouped for the syntheses.	Outlined in 'Methods'. Full eligibility criteria also provided in Supplementary Materials.
Information sources	6	Specify all databases, registers, websites, organisations, reference lists and other sources searched or consulted to identify studies. Specify the date when each source was last searched or consulted.	Outlined in 'Methods'.
Search strategy	7	Present the full search strategies for all databases, registers and websites, including any filters and limits used.	Outlined in 'Methods'. Full search strategies for each source also included in Supplementary Materials.
Selection process	8	Specify the methods used to decide whether a study met the inclusion criteria of the review, including how many reviewers screened each record and each report retrieved, whether they worked independently, and if applicable, details of automation tools used in the process.	Outlined in 'Methods'. Full eligibility criteria also included in Supplementary Materials.
Data collection process	9	Specify the methods used to collect data from reports, including how many reviewers collected data from each report, whether they worked independently, any	Outlined in 'Methods - Study selection, quality assessment, and data extraction' and 'Methods – Data Synthesis'.

Section and Topic	Item #	Checklist item	Location where item is reported
		processes for obtaining or confirming data from study investigators, and if applicable, details of automation tools used in the process.	
Data items	10a	List and define all outcomes for which data were sought. Specify whether all results that were compatible with each outcome domain in each study were sought (e.g. for all measures, time points, analyses), and if not, the methods used to decide which results to collect.	Outlined in 'Methods - Study selection, quality assessment, and data extraction' and 'Methods – Data Synthesis'. Data output in Table 1 with further details in Supplementary Materials Tables S1/S2.
	10b	List and define all other variables for which data were sought (e.g. participant and intervention characteristics, funding sources). Describe any assumptions made about any missing or unclear information.	Outlined in 'Methods - Study selection, quality assessment, and data extraction' and 'Methods – Data Synthesis'. Data output in Table 1 with further details in Supplementary Materials Tables S1/S2.
Study risk of bias assessment	11	Specify the methods used to assess risk of bias in the included studies, including details of the tool(s) used, how many reviewers assessed each study and whether they worked independently, and if applicable, details of automation tools used in the process.	Outlined in 'Methods' with references to the NOS templates. Complete assessments are included in Supplementary Materials.
Effect measures	12	Specify for each outcome the effect measure(s) (e.g. risk ratio, mean difference) used in the synthesis or presentation of results.	Outlined in Table 1 with further details in Supplementary Materials Tables S1/S2.
Synthesis methods	13a	Describe the processes used to decide which studies were eligible for each synthesis (e.g. tabulating the study intervention characteristics and comparing against the planned groups for each synthesis (item #5)).	Outlined in 'Methods - Study selection, quality assessment, and data extraction' and 'Methods – Data Synthesis'. Data output in Table 1 with further details in Supplementary Materials Tables S1/S2.
	13b	Describe any methods required to prepare the data for presentation or synthesis, such as handling of missing summary statistics, or data conversions.	Outlined in 'Methods - Study selection, quality assessment, and data extraction' and 'Methods – Data Synthesis'. Data output in Table 1 with further details in Supplementary Materials Tables S1/S2.
	13c	Describe any methods used to tabulate or visually display results of individual studies and syntheses.	Outlined in 'Methods - Study selection, quality assessment, and data extraction' and 'Methods – Data Synthesis'. Data output in Table 1 with further details in Supplementary Materials Tables S1/S2.
	13d	Describe any methods used to synthesize results and provide a rationale for the choice(s). If meta-analysis was performed, describe the model(s), method(s) to identify the presence and extent of statistical heterogeneity, and software package(s) used.	Outlined in 'Methods – Data Synthesis'. Referenced SWiM methodology and outlined.
	13e	Describe any methods used to explore possible causes of heterogeneity among study results (e.g. subgroup analysis, meta-regression).	Outlined throughout 'Results' and referenced 'SWiM' methodology in 'Methods'.
	13f	Describe any sensitivity analyses conducted to assess robustness of the synthesized results.	Outlined throughout 'Results' and referenced 'SWiM' methodology in 'Methods'.
Reporting bias assessment	14	Describe any methods used to assess risk of bias due to missing results in a synthesis (arising from reporting biases).	Outlined throughout 'Results' and referenced 'SWiM' methodology in 'Methods'. Details regarding quality

Section and Topic	Item #	Checklist item	Location where item is reported
			assessment tools i.e. NOS are referenced and outlined in 'Methods' with details for each study included in Supplementary Materials.
Certainty assessment	15	Describe any methods used to assess certainty (or confidence) in the body of evidence for an outcome.	Outlined throughout 'Results' and referenced 'SWiM' methodology in 'Methods'. Details regarding quality assessment tools i.e. NOS are referenced and outlined in 'Methods' with details for each study included in Supplementary Materials.
**RESULTS**			
Study selection	16a	Describe the results of the search and selection process, from the number of records identified in the search to the number of studies included in the review, ideally using a flow diagram.	Outlined in 'Results'. PRISMA Flow diagram also included – FIGURE 1.
	16b	Cite studies that might appear to meet the inclusion criteria, but which were excluded, and explain why they were excluded.	Details included in PRISMA Flow diagram and outlined in 'Results'. No ambiguity in studies appearing to meet inclusion criteria. Details regarding screening process/reviewers are included in 'Methods'.
Study characteristics	17	Cite each included study and present its characteristics.	Referenced throughout manuscript. Details included in Table 1 with further details in Supplementary Materials Tables S1/S2.
Risk of bias in studies	18	Present assessments of risk of bias for each included study.	Details included in Table 1 with further details in Supplementary Materials Tables S1/S2.
Results of individual studies	19	For all outcomes, present, for each study: (a) summary statistics for each group (where appropriate) and (b) an effect estimates and its precision (e.g. confidence/credible interval), ideally using structured tables or plots.	Details included in Table 1 with further details in Supplementary Materials Tables S1/S2.
Results of syntheses	20a	For each synthesis, briefly summarise the characteristics and risk of bias among contributing studies.	Referenced throughout Results.
	20b	Present results of all statistical syntheses conducted. If meta-analysis was done, present for each the summary estimate and its precision (e.g. confidence/credible interval) and measures of statistical heterogeneity. If comparing groups, describe the direction of the effect.	Outlined methods for data synthesis in 'Methods' with reference to 'SWiM' methodology. Meta-analysis was not conducted.
	20c	Present results of all investigations of possible causes of heterogeneity among study results.	Outlined throughout Results as per 'SWiM' methodology.
	20d	Present results of all sensitivity analyses conducted to assess the robustness of the synthesized results.	Discussed throughout Results.
Reporting biases	21	Present assessments of risk of bias due to missing results (arising from reporting biases) for each synthesis assessed.	Quality of study assessments included in Table 1 with further details in Supplementary Materials Tables S1/S2. SWiM methodology used for reporting.
Certainty of evidence	22	Present assessments of certainty (or confidence) in the body of evidence for each outcome assessed.	Quality of study assessments included in Table 1 with further details in

Section and Topic	Item #	Checklist item	Location where item is reported
			Supplementary Materials Tables S1/S2. SWiM methodology used for reporting. Meta-analysis was not conducted.
**DISCUSSION**			
Discussion	23a	Provide a general interpretation of the results in the context of other evidence.	Outlined in 'Discussion' under relevant sub-headings also.
	23b	Discuss any limitations of the evidence included in the review.	Outlined in separate 'Limitations' section.
	23c	Discuss any limitations of the review processes used.	Outlined in separate 'Limitations' section.
	23d	Discuss implications of the results for practice, policy, and future research.	Outlined in 'Conclusions'.
**OTHER INFORMATION**			
Registration and protocol	24a	Provide registration information for the review, including register name and registration number, or state that the review was not registered.	Provided in 'Methods'. [PROSPERO (CRD420251063020)].
	24b	Indicate where the review protocol can be accessed, or state that a protocol was not prepared.	Protocol was registered. [PROSPERO (CRD420251063020)].
	24c	Describe and explain any amendments to information provided at registration or in the protocol.	Nil amendments.
Support	25	Describe sources of financial or non-financial support for the review, and the role of the funders or sponsors in the review.	Nil funding.
Competing interests	26	Declare any competing interests of review authors.	Nil COI.
Availability of data, code and other materials	27	Report which of the following are publicly available and where they can be found template data collection forms; data extracted from included studies; data used for all analyses; analytic code; any other materials used in the review.	Data extracted is included in Table 1 with further details in Supplementary Materials Tables S1/S2. SWiM methodology used for reporting is referenced. Quality of Assessment methodology is referenced, with quality assessments for each study included in Table 1 with further details in Supplementary Materials. All studies are referenced.
